# Methodological Choices Strongly Modulate the Sensitivity and Specificity of Lesion‐Symptom Mapping Analyses

**DOI:** 10.1002/hbm.70619

**Published:** 2026-07-30

**Authors:** Margaret Jane Moore, Chris Rorden, Gail A. Robinson, Jason B. Mattingley, Nele Demeyere

**Affiliations:** ^1^ Queensland Brain Institute, University of Queensland St. Lucia Queensland Australia; ^2^ Department of Psychology University of South Carolina Colombia South Carolina USA; ^3^ School of Psychology, University of Queensland Brisbane Queensland Australia; ^4^ Canadian Institute for Advanced Research Toronto Ontario Canada; ^5^ Nuffield Department of Clinical Neurosciences University of Oxford Oxford UK

**Keywords:** lesion‐symptom mapping, multivariate lesion mapping, multiverse analysis, neuroimaging, stroke

## Abstract

Lesion‐symptom mapping results can vary substantially as a function of specific analysis parameters, but the extent to which individual methodological choices interact to modulate the sensitivity and specificity of results is not clear. Here, we employed a large‐scale simulation approach to inform practical recommendations for lesion‐symptom mapping studies. Routine clinical imaging from 959 stroke survivors (mean age = 72.5, 49.3% female) was used to conduct 384,780 lesion‐symptom mapping analyses based on simulated behavioural data. Each simulated analysis used different combinations of plausible sample inclusion criteria, analysis parameters (e.g., correction factors), analysis types (e.g., univariate vs. multivariate), and underlying target correlates. Simulated analysis accuracy (percent coverage of target correlates, Dice similarity coefficient, and false positives) was compared across designs. Overall, analysis accuracy varied widely and was substantially modulated by the specific design used. Analyses that maximised lesion coverage by including large and diverse samples reliably outperformed analyses using more restricted samples. Analyses using direct total lesion volume controls outperformed analyses using other (or no) volume corrections across all accuracy measures. False discovery rate corrections yielded the best performance in terms of target coverage, while permutation corrections yielded the best Dice coefficients. While multivariate approaches were more accurate than univariate analyses in terms of Dice coefficient, univariate analyses generated higher target hit rates and percent target coverage. These results identify specific analysis designs suitable for studies aiming to maximise their sensitivity and/or specificity to underlying critical correlates, while highlighting the inferential strengths and weaknesses of these complementary approaches.

## Graphical abstract

Lesion mapping accuracy is strongly modulated by specific methodological choices. This study identifies specific analysis designs suitable for studies aiming to maximise their sensitivity and/or specificity to underlying critical correlates, while highlighting the inferential strengths and weaknesses of these complementary approaches.

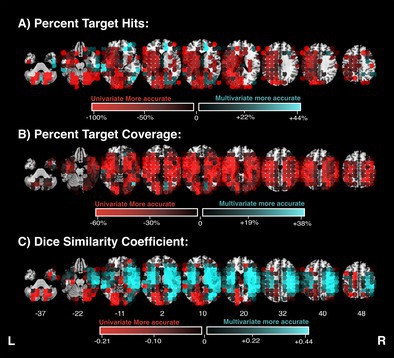

## 1 Introduction

Lesion‐symptom mapping is a popular analytical approach in which the location of brain damage is used to infer causal brain‐behaviour relationships (Bates et al. 2003; de Haan and Karnath 2018; Moore, Demeyere, et al. 2023). This method identifies brain regions that are associated with impairments when injured and associated with intact function when spared (Bates et al. 2003; de Haan and Karnath 2018; Moore, Demeyere, et al. 2023; Rorden and Karnath 2004). Lesion‐symptom mapping methods have been implemented to localise the neural correlates of a wide range of fundamental cognitive functions (Moore et al. 2024; Weaver et al. 2021). However, recent research has provided evidence that the accuracy of unconstrained lesion‐symptom mapping analyses may be spatially biased (Inoue et al. 2014; Mah et al. 2014), and that results can vary substantially as a function of specific analysis choices (Ivanova et al. 2021; Mirman et al. 2018; Sperber et al. 2019). The aim of the current study was to use a large‐scale simulation approach to extend understanding of how specific analytical choices interact to modulate the outcomes of lesion‐symptom mapping analyses.

Lesion‐symptom mapping, like any other statistical approach, entails an inherent trade‐off between sensitivity and specificity (Kimberg et al. 2007; Sperber 2022). In lesion‐symptom mapping, sensitivity can be defined as the proportion of identified significant voxels which overlap with underlying ground‐truth correlate, divided by the number of voxels included in the relevant ground‐truth correlate. Specificity can be defined as the number of voxels outside the ground‐truth correlate successfully classified as non‐significant, divided by the total number of considered voxels outside the ground‐truth correlate. Lesion‐symptom mapping analyses aiming to optimize sensitivity may employ more liberal statistical approaches to prioritize the detection of significant neural correlates over minimizing the risk of voxel‐wise false positives (Sperber 2022). Analyses aiming to maximise specificity may opt for more conservative methods to identify “core” correlates over minimizing the risk of false negative results (Moore et al. 2024; Moore and Demeyere 2022). Alternatively, researchers may wish to take a balanced statistical approach. Each of these methodological choices is well‐suited to addressing specific types of research questions in lesion‐symptom mapping analyses, but the specific.

When planning any lesion‐symptom mapping analysis, researchers must select from many design options which each have the potential to modulate sensitivity and specificity. For example, researchers need to choose sample inclusion/exclusion criteria by considering (or not considering) factors such as location of the lesion, imaging modality, and lesion numerosity (de Haan and Karnath 2018; Moore, Jenkinson, et al. 2023). Each of these choices has potentially critical consequences. For example, the spatial distribution (and degree of overlap) of lesions within study samples constrains the range of areas analyses are able to draw conclusions about (Moore, Jenkinson, et al. 2023). Researchers must also select between an extensive range of potential statistical approaches (e.g., univariate versus multivariate), covariate controls, multiple comparison corrections, and minimum lesion overlap inclusion criteria (Ivanova et al. 2021; Mirman et al. 2018; Sperber and Karnath 2017). Simulation‐based lesion‐symptom mapping approaches have been applied to provide insight into the impact of analysis design choices on analysis accuracy (Ivanova et al. 2021; Mah et al. 2014; Moore and Demeyere 2022; Sperber et al. 2019). In this approach, many potential datasets and corresponding analysis designs can be generated by making each possible permutation of reasonable methodological choices and underlying ground‐truth targets (Dragicevic et al. 2019; Ivanova et al. 2021). Each of these potential experimental designs can then be simulated, allowing for cumulative results to be evaluated to identify factors leading to results variability.

For example, previous studies have used this approach to compare the accuracy of univariate versus multivariate lesion‐symptom mapping approaches (Ivanova et al. 2021; Mah et al. 2014; Moore and Demeyere 2022; Sperber et al. 2019). While traditional lesion‐symptom mapping approaches generally use mass‐univariate statistical approaches, multivariate approaches are rising in popularity (DeMarco and Turkeltaub 2018; Moore, Demeyere, et al. 2023; Pustina et al. 2018; Zhang et al. 2014). Mass‐univariate approaches consider each voxel independently and are therefore theoretically unable to distinguish between critical neural correlates and non‐critical areas that are likely to be damaged by the same lesions (Mah et al. 2014). Multivariate approaches may potentially address this limitation by considering all voxels concurrently to generate a single, robust prediction of impairment, but the evidence in support of this has not been consistent across all studies (DeMarco and Turkeltaub 2018; Mah et al. 2014; Moore, Demeyere, et al. 2023; Pustina et al. 2018).

Mah et al. (2014) used a simulation approach in a large sample (*n* = 581) to quantify spatial mislocalisation in univariate lesion‐symptom mapping analyses, and to propose multivariate approaches to ameliorate this issue. Similarly, Pustina et al. (2018) used simulation approaches to demonstrate that multivariate approaches result in higher agreement between significant voxels and target voxels (Dice coefficient), and reduce the spatial displacement of results relative to univariate analyses. However, this finding has not been consistent across all simulation studies. Sperber et al. (2019) applied a simulation approach to measure the accuracy of univariate versus multivariate lesion‐symptom mapping and to identify factors that modulate the accuracy of multivariate lesion‐symptom mapping. The results of that study suggest that multivariate lesion‐symptom mapping approaches are susceptible to a similar degree of spatial displacement as univariate approaches, and that large samples (*n* = 100+) and corrections for multiple comparisons are needed for accurate multivariate analyses (Sperber et al. 2019). This result was replicated in a subsequent, more comprehensive simulation study (Ivanova et al. 2021). Additional simulation work has indicated that the accuracy of multivariate approaches also appears to be highly variable when different analysis parameters (e.g., lesion volume) and sample sizes are used (DeMarco and Turkeltaub 2018; Sperber et al. 2019). Notably, the accuracy of multivariate analyses is also often quantified in terms of impairment prediction accuracy, rather than the extent to which resulting significant voxels align with underlying “true” neural correlates (Griffis et al. 2023). However, robust impairment predictions can be supported by voxels that are not underlying critical impairments (Griffis et al. 2023; Moore, Demeyere, et al. 2023). For this reason, additional work specifically quantifying the degree of agreement with underlying ground‐truth neural correlates is needed to clarify what the optimal statistical approach for lesion‐symptom mapping is.

Additional simulation work has identified analysis parameters outside of the type of statistical tests used as key factors modulating the accuracy of lesion‐symptom mapping results. For example, Sperber (2022) conducted a lesion‐symptom mapping simulation analysis which showed that including lesion volume as a covariate of no interest reduces the false positive rate in lesion‐symptom mapping, but results in a corresponding drop in sensitivity. Moreover, additional work has demonstrated that the magnitude of false positive rate reduction differs across different volume correction types, with some types of volume corrections argued to be too conservative (DeMarco and Turkeltaub 2018; Zhang et al. 2014). Applying lesion volume corrections results in higher specificity, which may be in line with the goals of some, but not all, study designs (Moore et al. 2024; Sperber 2022).

Mirman et al. (2018) applied a simulation approach and found that lesion‐symptom mapping analyses using False Discovery Rate (FDR) corrections (relative to more conservative permutation corrections) generate very liberal results which may be spatially displaced from true, underlying neural correlates. However, the results of that study suggest that corrections for multiple comparisons and lesion volume significantly interact to modulate analysis accuracy. Studies aiming to prioritize specificity generally opt for either family‐wise error rate via permutation or Bonferroni corrections, but these approaches may be too conservative (de Haan and Karnath 2018; Mirman et al. 2018). Lesion‐symptom mapping analyses using Bonferroni, permutation, and FDR corrections have not yet been directly compared in terms of sensitivity and specificity. There is a similar lack of consensus surrounding voxel‐level minimum inclusion criteria, with different studies arguing the advantages and disadvantages of restricting analysis to voxels impacted by sufficient lesions (e.g., 10% of the total sample) (Sperber and Karnath 2017). Sperber and Karnath (2017) used a simulation approach to demonstrate that lesion‐symptom mapping accuracy is higher when analyses are constrained to voxels affected in at least 5 patients, relative to designs in which all impacted voxels are included. It remains unclear, however, whether analysis accuracy improves as a linear function of the minimum overlap threshold employed, or whether there is an optimum proportion of voxels to retain in lesion‐symptom mapping analyses.

Notably, Ivanova et al. (2021) reported a comprehensive simulation study exploring how several methodological factors, including sample size, anatomical target location/size, degree of lesion mask smoothing, and noise in simulated behaviour, interact to modulate analysis accuracy. In this simulation, sample size emerged as a key factor, with results accuracy quantified in terms of spatial displacement improving as sample size increased. Correspondingly, accuracy was lowest at anatomical targets with lower levels of lesion overlap (Ivanova et al. 2021). The degree of results and anatomical target overlap was modulated by target size, with larger targets yielding more accurate results. Additionally, behavioural noise was found to reduce analysis accuracy (Ivanova et al. 2021). Overall, Ivanova et al. (2021) concluded that both univariate and multivariate lesion‐symptom mapping approaches can yield results that are spatially displaced from the underlying “ground‐truth” target, but the magnitude of this displacement is also modulated by other analysis parameters.

Despite this body of previous work, several key questions remain unexplored. In real‐world analyses, the distribution of included lesions is ultimately a product of the sample inclusion criteria used. This means that inclusion criteria such as impacted hemisphere, stroke numerosity, and imaging modality may result in systematically different lesion distributions which may, in turn, modulate the accuracy of lesion‐symptom mapping analyses. While previous experiments have explored the impact of sample size in lesion‐symptom mapping by randomly selecting a specific number of lesions to include (Ivanova et al. 2021), it remains unclear whether those findings will be replicated when the number and distribution of included lesions are determined by realistic potential inclusion criteria. Additionally, past studies have aimed to maximise sample sizes by combining different neuroimaging modalities and including comparatively low‐resolution routine clinical imaging (e.g., CT). However, the benefits and drawbacks of this approach relative to study designs that employ exclusively higher resolution neuroimaging (e.g., MRI) have not yet been systematically evaluated (de Haan and Karnath 2018). Additionally, the benefit of alternative methods for lesion volume correction (such as direct total lesion volume) has not yet been quantified in simulation studies.

Here we employed a simulation‐based approach to address this key knowledge gap and to support practical recommendations for lesion‐symptom mapping studies aiming to optimize outcomes. Specifically, we aimed to identify sample inclusion criteria (stroke side, scan type, stroke numerosity), analysis parameters (minimum overlap threshold, multiple comparison corrections, lesion volume corrections), and statistical approach (chi‐squared, regression, sparse canonical correlation) which modulate the extent to which simulated lesion‐symptom mapping analyses can identify ground‐truth underlying correlates. Analysis accuracy was quantified in terms of proportion of target hits, proportion of target voxels classed as significant, and degree of agreement between binarized target and lesion‐symptom mapping results (Dice coefficient). The findings provide a highly detailed insight into how the choices made in lesion‐symptom mapping designs ultimately impact accuracy and offer guidance for future lesion‐symptom mapping studies.

## 2 Methods

### 2.1 Stroke Neuroimaging Data

We report a retrospective analysis of existing routine stroke neuroimaging data collected as a component of the Oxford Cognitive Screening Program (NHS REC reference 14/LO/0648, 18/SC/0550, 12/WM/00335) and by The University of Queensland's Neuropsychology Clinic (Metro South and Metro North Queensland Health and the University of Queensland HREC/16/QPAH/793). Participants were included in the present study if they had clinical imaging depicting visible stroke lesions and that the scan was of sufficient quality to support lesion delineation and spatial normalisation. All participants provided informed written consent (in line with the Declaration of Helsinki). Data from 959 stroke patients (mean age = 72.5 (SD = 13.3), 49.3% female, 8.0% left‐handed) were included in the present study.

Lesion masks were generated in line with the standard processing protocol reported by Moore and Demeyere (2022) (voxel size = 1 × 1 × 1 mm^3^). Specifically, lesions were manually delineated on native space axial scan slices by a trained expert (MJM) (765 CT, 194 MR) (135 T2, 1 T1, 58 FLAIR). This imaging was collected a mean of 14.4 days post stroke (SD = 68.2, range = 0–924). Stroke types were reported as 61.4% Ischemic, 33.3% Haemorrhagic, and 5.3% unreported, and lesion sides were reported as 37.8% left, 50.2% right, 18.3% bilateral.

Notably, 57/959 (5.94%) of included brain scans were collected greater than 30 days post‐stroke (mean = 166.3, SD = 188.6, range = 31–924 days). It is not recommended to combine imaging collected in acute versus non‐acute stages post stroke as neural recovery can add potentially confounding noise to analyses localizing brain‐behaviour relationships (Karnath et al. 2011; Moore, Demeyere, et al. 2023). However, this issue is not relevant to the present study as all behaviour is simulated. Additionally, supplementary analyses indicate that the lesion distributions yielded by acute versus non‐acute imaging in this study are comparable in terms of severity and distribution (Figure S1).

Masks were smoothed at 5 mm full width at half maximum in the z‐direction and binarised using MRIcron (Rorden 2007). Scans and lesions were then reoriented to the anterior commissure to reduce transformation degrees of freedom and warped into 1 × 1 × 1 mm MNI space using nonlinear normalisation and age‐matched templates (SPM, Ashburner et al. 2016; Clinical Toolbox, Rorden et al. 2012). Normalised scans and lesions were visually inspected for accuracy prior to inclusion. All scans that could not be accurately normalised were excluded.

### 2.2 Simulation Analysis

The voxels underlying real‐world neuropsychological deficits are ultimately unknown, meaning that simulation‐based lesion‐symptom mapping approaches are required to measure analysis accuracy (Ivanova et al. 2021; Mah et al. 2014; Moore and Demeyere 2022). In this approach, simulated behavioural scores are generated from real lesion data by defining the extent to which each considered lesion overlaps with a pre‐defined critical target site (e.g., target ROIs). These critical sites include regions that are commonly impacted in vascular‐related neurological injuries, as well as regions that are less commonly damaged in stroke. These simulated behavioural scores can then be input into lesion‐symptom mapping analyses, allowing for accuracy to be quantified by comparing the resultant significant voxels to the known target site.

This approach was used in two separate simulation analyses (univariate and multivariate) to investigate how different analysis parameters modulate results accuracy.

**FIGURE 1:**
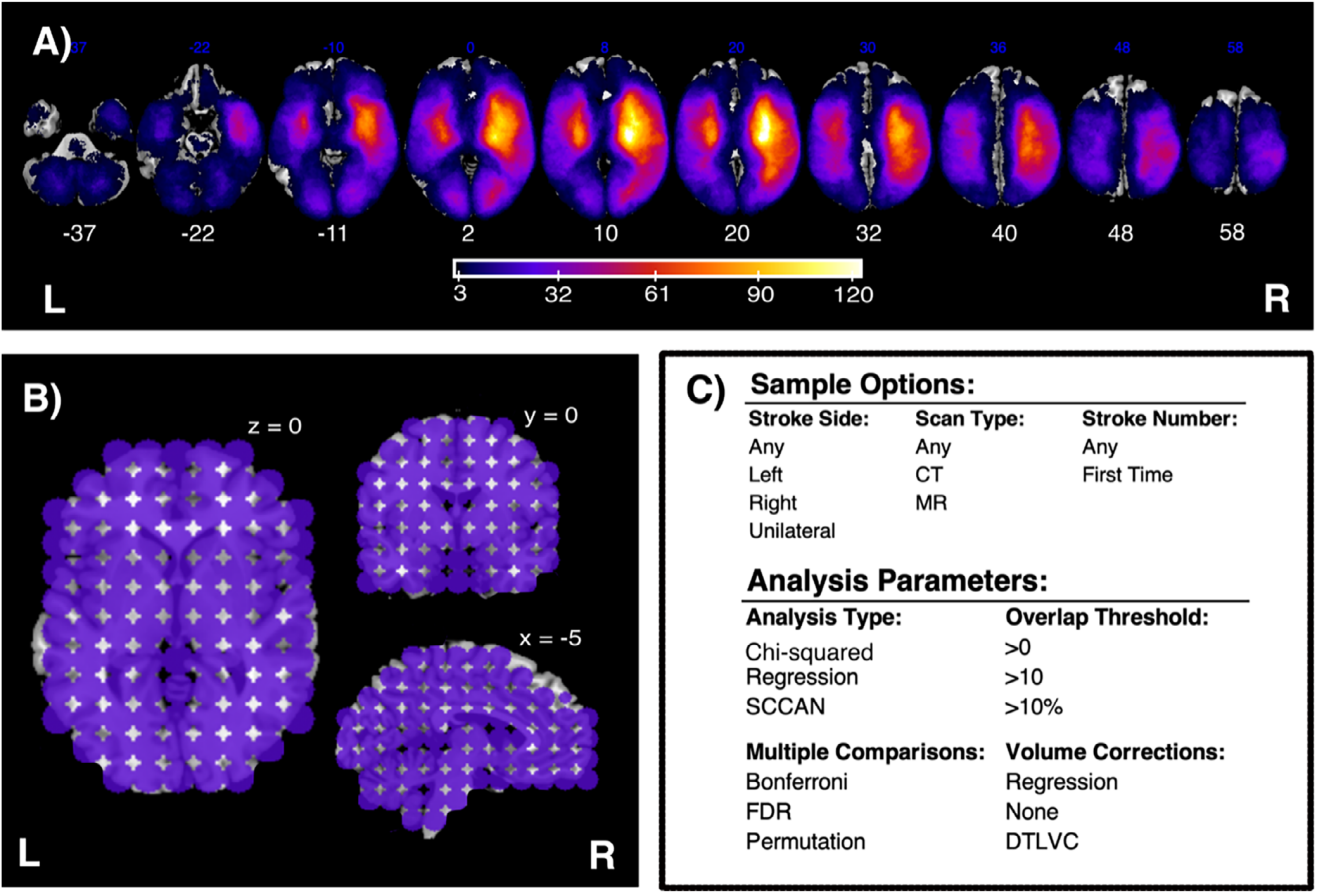
A visualisation of the lesion data and parameters used in this study's simulation analyses. (A) Lesion overlay plot for the full sample (*n* = 959). Colour denotes number of overlapping lesions at each location. Only areas impacted in at least 3 lesions are visualised. MNI slices 37–58 are presented. (B) The anatomical targets used in this study. Only targets impacted in at least three lesions and falling within the selected MNI slices are included in this visualisation. (C) All patient sample and analysis parameter options considered in the univariate analyses. The specific parameters considered in multivariate simulations are reported in text.

Target ROIs were generated by creating an array of spheres (volume = 4.2 cm^3^) covering the brain (Figure 1B). Arbitrarily defined target shapes and locations were used to standardise the size and shape of underlying target regions and to maximise coverage of the brain. This approach was followed to ensure balance between the detail and scope of previous studies that used comprehensive, single‐voxel targets (Mah et al. 2014; Moore, Jenkinson, et al. 2023) and studies that used a select number of naturalistic ROIs as targets (Ivanova et al. 2021; Sperber et al. 2019). The employed target volume was arbitrarily selected to ensure adequate coverage while balancing the computational resources needed to evaluate performance at each target. Real patient lesion masks were compared with each target to simulate impairment severities (the percent of target ROI voxels impacted by each lesion). Targets impacted by fewer than 3 lesions were not included in analyses, resulting in 671 target areas. This study reports accuracy measures derived from considering the degree of overlap between all significant voxels and the underlying target regions. However, accuracy results which consider only the voxels yielding peak statistical values are reported in Supporting Information (Figure S2, Table S1).

Simulation analysis accuracy was quantified using three metrics: percent target coverage, Dice coefficient, and false positive rate. Multiple accuracy metrics were used as each measure captures some, but not all, aspects associated with the degree of alignment between results and underlying true correlates. Percent target coverage is the proportion of target ROI voxels which are significant in simulated analysis. Percent coverage captures sensitivity (i.e., false negative rate) and does not penalise based on false positive rate (i.e., target overestimation). Conversely, false positive rate is the proportion of significant voxels that are not target voxels, providing a measure of voxel‐wise specificity. Dice coefficient provides a standard measurement of the agreement between two binary image segmentations, which penalises voxel masks for both over‐ and under‐estimating the underlying target area. This measure was included as it is a standard approach and is the main accuracy measure used in past lesion‐symptom mapping simulation studies (e.g., Ivanova et al. 2021). Dice captures both voxel‐wise sensitivity and specificity. Percent coverage and Dice have a U‐shaped relationship, such that low Dice coefficients occur both when percent coverage is very high (prone to overestimation) and very low (underestimation) (Figure S3).

All simulation analyses were implemented using LESYMAP (Pustina 2019) in R and were run using The University of Queensland's High Performance Computing Cluster (Dell EMC Technologies Australia Pty Ltd).

### 2.3 Univariate Simulation Analyses

The purpose of the univariate simulation analyses was to investigate how different sample inclusion criteria and analysis parameters modulate the accuracy of lesion‐symptom mapping analyses. Within these analyses, behavioural scores were simulated using the approach described above but the patients included in each analysis (sample inclusion criteria) and the lesion‐symptom mapping approach used (analysis parameters) were systematically varied across simulated analyses.

Possible sample inclusion criteria included all possible combinations of stroke side, scan type, and stroke history inclusion criteria (Figure 1C). Within stroke side, *Left/Right* categories included all patients with damage confined to the relevant brain hemisphere, *Unilateral* included patients with lesions confined to a single hemisphere, and *unselected* include all patients, regardless of lesion location. This manipulation was included as some past lesion mapping studies restrict samples to patients with damage to a specific hemisphere while others do not and it is plausible that this may differentially introduce spatial biases into lesion‐symptom mapping results (Moore, Milosevich, et al. 2023). Within scan type, *CT/MR* included patients with the relevant imaging type while *Unselected* included all patients regardless of imaging modality. This variable was included to explore how sample characteristics (and results accuracy) differ as a function of imaging modality requirements (Moore, Jenkinson, et al. 2023). Within stroke history, *First Stroke* included only patients exhibiting evidence of a single stroke event while *Unselected* analyses included all patients regardless of stroke numerosity. Given the large scale of this study (*n* = 959), these inclusion criteria generally yielded large sample sizes in individual analyses (mean *n* = 463, range = 67–959). This sample size range is comparable to previous large‐scale lesion‐symptom mapping studies but is substantially larger than many smaller‐scale lesion‐symptom mapping designs (e.g., *n* = 20–100) (Bowren Jr et al. 2022; Huygelier et al. 2025; Levy et al. 2024; Moore et al. 2024). This approach was adopted to prioritise findings which can be generalised to larger‐scale lesion‐symptom mapping designs (e.g., samples > 100), as a minimum sample size of 100 is recommended in lesion‐symptom mapping (Moore, Demeyere, et al. 2023; Sperber et al. 2019).

Analysis parameter options were generated by varying analysis type, multiple comparison corrections, minimum overlap threshold, and lesion volume corrections. Within univariate simulation analysis types, Chi Squared (with Yates correction) and regression analyses were used. Chi Squared analyses test binary associations between voxel impairment (spared/impaired) and behavioural scores (spared/impaired). Within these analyses, lesions which overlapped with the relevant behavioural target were scored as impaired while all other patients were considered unimpaired. Chi‐squared analyses were included as the binary analysis option in this study as other common approaches (e.g., Liebermeister tests) are not implemented in LESYMAP (Pustina 2019; Rorden et al. 2009). In cases where voxel values are binary (spared/impaired), the regression analyses used are equivalent to non‐parametric *t*‐tests but have the added benefit of allowing voxel‐level corrections for multiple comparisons (described below) (Pustina 2019). In cases where these corrections are used, the regression approach correlates continuous behavioural scores with volume‐corrected lesion values (Pustina 2019).

Three different multiple comparison corrections were applied (Bonferroni, 5% False Discovery Rate (FDR), and family‐wise error rate permutation). Bonferroni corrections represent a very strict correction approach in which the baseline significance threshold (alpha = 0.05) is lowered by dividing this value by the number of conducted voxel‐level analyses (Bonferroni‐corrected alpha = 0.05/number of tests per simulated analysis). FDR corrections represent a less stringent control which determines the corrected alpha level to allow a specific proportion of expected false positive voxel‐level results relative to the total number of significant results (Mirman et al. 2018). In the current study, a 5% FDR correction was applied. Family‐wise error rate permutation corrections determine significance thresholds by empirically simulating null distributions of the maximum test statistic yielded by analyses with randomly permuted behavioural scores (Pustina et al. 2018). We used 1000 voxel‐level permutations to generate this null distribution, and considered test statistics exceeding the 95th percentile of the null distribution to be significant.

Three minimum overlap thresholds (voxels impacted in at least one patient (> 0), voxels impacted in at least 10 patients (> 10), and voxels impacted in at least 10% of the sample) were considered. Within lesion volume corrections, *None* analyses employed no correction for lesion volume, *Regression* analyses included volume as a covariate of no interest in analysis, and *DTLVC* analyses employed direct total lesion volume control corrections (DTLVC, Zhang et al. 2014). DTLVC normalises binary voxel scores (0 = spared, 1 = lesioned) by the square root of each patient's lesion volume, implementing a less conservative correction than regressing lesion volume from behavioural scores (Zhang et al. 2014). Notably, DTLVC does not apply this correction to voxels which are not lesioned. When impossible analysis parameters combinations were excluded (e.g., Chi‐squared analyses with volume corrections), these analysis parameters yielded 33 unique analysis designs. Each of these potential analysis designs was applied for each target ROI (*n* = 759) and sample groups (*n* = 24) yielding 601,128 unique potential univariate simulation analyses.

The results generated from these simulated lesion‐symptom mapping analyses were then cumulatively analysed to measure the extent to which analysis accuracy (quantified in terms of percent target hits, Dice, and percent coverage) varied across different analysis designs. Specifically, accuracy was compared across underlying anatomical targets.

Linear mixed effects models were used to determine the extent to which general analysis factors (e.g., sample size, average lesion volume, number of lesions impacting the target, results cluster size) modulated accuracy. These models included analysis factors as fixed effects, and underlying target identity as a random effect. Parameter‐specific *p*‐values were generated using Satterthwaite approximation. Multivariate regression analyses were conducted to evaluate whether individual analysis designs modulated accuracy when general analysis factors were considered. In these analyses, the use of each individual analysis parameter (binarised) was added to a multivariate regression predicting results accuracy from general analysis factors. In cases where added analysis choices significantly and positively explained accuracy variance when considered alongside general analysis factors, these analysis choices were considered to be associated with improved accuracy. Throughout the Results section, all reported *R* ^2^ values are adjusted. Unless otherwise stated, all reported *R* ^2^ values are marginal *R* ^2^ values for linear mixed modelling analyses and adjusted for multivariate regression results.

### 2.4 Multivariate Simulation Analyses

Next, multivariate simulation analyses were conducted to allow for the accuracy of multivariate and univariate lesion‐symptom mapping approaches to be compared. Specifically, the accuracy of simulated sparse canonical correlation‐based lesion‐symptom mapping (SCCAN) was compared with that of matched univariate simulations (described above). SCCAN is a popular multivariate lesion‐symptom mapping approach that builds a best‐fit model leveraging multivariate lesion patterns to predict behavioural scores. The voxels retained in this model represent the predicted neural correlates associated with behavioural scores (Pustina et al. 2018). SCCAN uses a modified L1 penalty to handle highly correlated input variables (Pustina et al. 2018). The percentage of input predictors included in final SCCAN solutions was optimised through an iterative sparseness optimisation and cross‐validation procedure (Pustina et al. 2018). Using this approach, SCCAN models are considered significant if a model's predicted behavioural scores are above chance (*p* < 0.05) within cross‐validation.

Notably, this sparseness optimisation procedure is computationally intensive, meaning that it is not feasible to simulate all possible analyses. This issue increases exponentially as the number of voxels (and lesion masks) included in the analysis increases (Pustina et al. 2018). This issue was managed by reducing the number of factors considered in multivariate simulations (relative to univariate simulations). First, to reduce the maximum sample size and number of voxels considered, only patients with unilateral stroke damage (i.e., exclusively left or right hemisphere damage) were included in multivariate simulations, yielding two possible sample groups. All other sample inclusion criteria (e.g., scan type, stroke number) were not considered in multivariate simulations. In terms of analysis parameters, potential minimum overlap thresholds were reduced to > 10 and 10% to further minimize the number of voxels considered in analysis. Volume correction options were reduced to *regression* and *no corrections*, as DLVTC corrections cannot be implemented in SCCAN analyses (Pustina 2019). SCCAN does not employ corrections for multiple comparisons, and voxel‐wise significance is dependent on model fit (Pustina et al. 2018). These factors yielded 8 possible analysis designs which were applied over the 759 behavioural targets, resulting in 6072 potential simulated multivariate lesion‐symptom mapping analyses. The data were used to evaluate the relative accuracy of multivariate approaches by comparing the results of these simulations with those of univariate approaches using matched analysis parameters and samples.

### 2.5 Pre‐Registration and Data Availability

This study's simulation analysis design and core analyses were pre‐registered on the Open Science Framework (https://osf.io/6kcha/registrations). The final study procedure deviated from the pre‐registered protocol in that only a subset of possible analysis types was explored in the multivariate simulation analyses. As noted above, this change was made to ensure the computational resources and processing time needed to run the simulations remained practical. All pre‐registered analyses were conducted in line with the registered procedure. Non‐registered exploratory analyses are identified as such in the relevant Results section. All code and simulated data associated with the project are openly available on the Open Science Framework (https://osf.io/6kcha/). Lesion masks are available on request from the authors.

## 3 LSM Multi Results

### 3.1 Univariate Analyses General Descriptives

63.4% of the potential univariate analysis designs met criteria for inclusion in the simulation analysis. Of these analyses, 72.0% yielded significant results, 66.7% of which overlapped with the underlying target. The average significant voxel cluster size was 11.5 cm^3^ (SD = 16.1, 0.001–130.4). Within analyses yielding significant results, the average target coverage was 43.6% (SD = 41.6, range = 0–100), the average Dice was 0.05 (SD = 0.08, range = 0–0.73), and the average false positive rate was 96.2% (SD = 0.08, range = 0–100).

**FIGURE 2:**
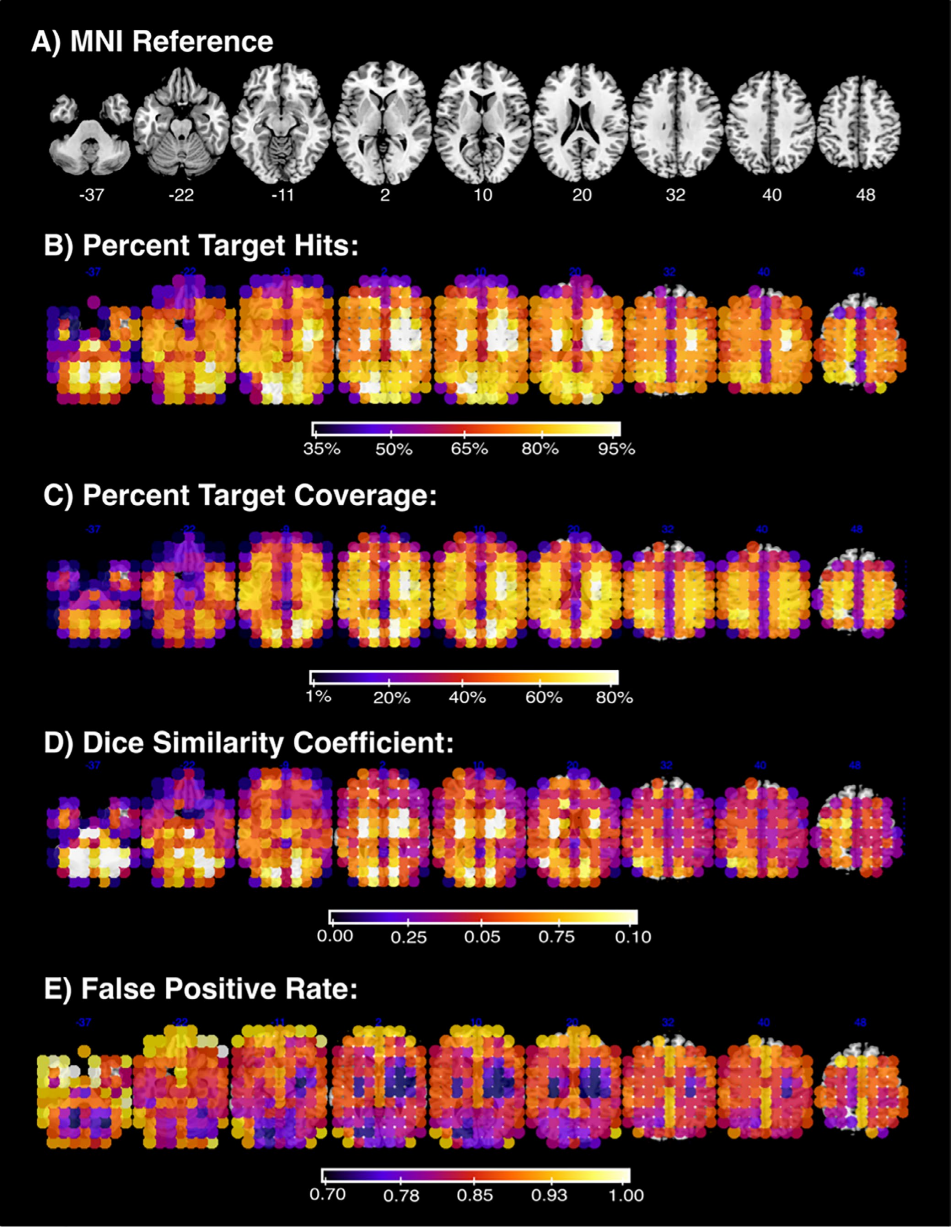
Lesion‐symptom mapping accuracy across different anatomical target areas. (A) Provides a MNI reference to aid interpretation of subsequent panels. (B) The percent of analyses which yielded significant results overlapping with the target for each considered anatomical target. (C) The percent of target voxels which were identified as significant for each considered anatomical target. (D) Dice similarity coefficient between significant voxels and underlying anatomical target voxels. (E) False positive rate of analyses using each anatomical target. Colour denotes mean accuracy for all analyses employing each defined anatomical target. Summary statistics reported at each target only include data from simulated analyses using the relevant target to simulate behaviour. L = left, R = right.

Analysis accuracy varied across the target ROIs (Figure 2). Of the analyses producing significant results, the probability of hitting the underlying target roughly aligned with the degree of statistical power present in the lesion distribution. Hit probability was highest within the MCA territory (particularly subcortical regions) and lowest in regions less commonly impacted in the sample (e.g., the most anterior frontal regions, peripheral cerebellar regions, and regions spanning the midline) (Figure 2B). This accuracy distribution was mirrored in the average target coverage, with very high coverage in areas with high lesion overlap (particularly subcortical regions) and poor accuracy in less affected areas (Figure 2C). A similar pattern emerged in results false positive rate, with lower false positive rates at areas with higher levels of lesion coverage (Figure 2E). Results Dice was highest within the pons, bilateral basal ganglia, and cerebellum (Figure 2D). Each of these regions is commonly impacted by small, relatively spatially homogeneous lesions (e.g., basal ganglia lacunar strokes) (Bassetti et al. 1996; Wardlaw 2005).

**FIGURE 3:**
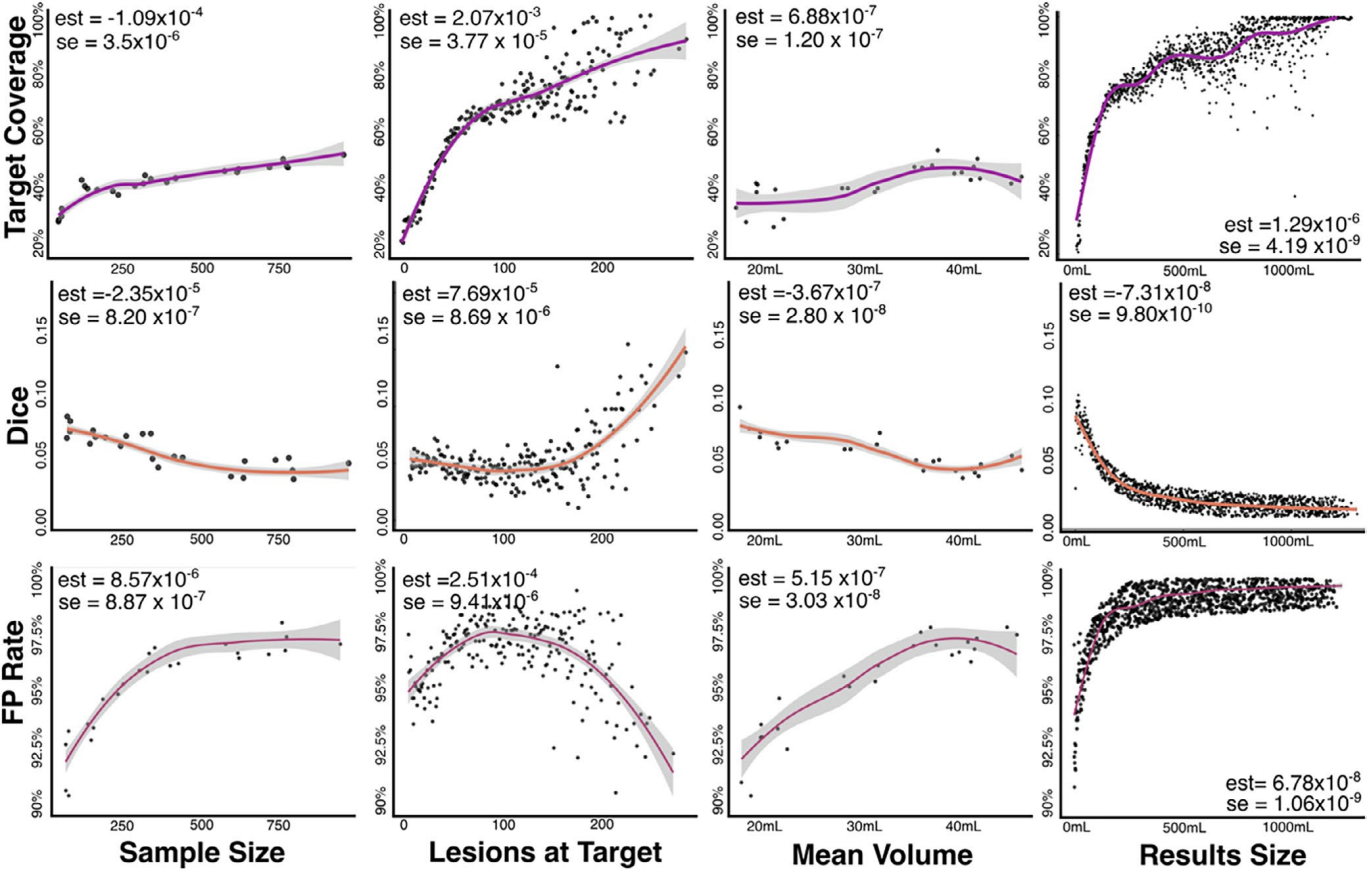
The relationship between key sample/results characteristics and analysis accuracy. Considered analysis factors are listed across the *x*‐axis, while different accuracy measures (Coverage, Dice, false positive (FP) rate) are on the *y*‐axis. The estimates and standard errors for each factor within the described linear mixed models are listed in the top left of each panel, while each panel depicts the best‐fit general linear model for each individual factor.

Across all univariate analyses yielding significant results, multivariate regression revealed that results accuracy (in terms of coverage, Dice, and false positive rate) was significantly modulated by sample size, average sample lesion volume, number of lesions impacting the target, and significant results cluster size (Coverage Model *R* ^2^ = 0.322, Dice Model: *R* ^2^ = 0.037, Percent False Positive Rate Model: *R* ^2^ = 0.052) (see Figure 3). All analysis factor fixed‐effect *p*‐values are < 0.001. Linear mixed model fixed estimates, standard error, and *p*‐values are reported in Figure 3.

While accuracy quantified in terms of percent coverage increases as sample size grows, Dice decreases and false positive rate increases (Figure 3). However, all accuracy measures improved as the number of lesions impacting the ground‐truth target increased (Figure 3). While the number of lesions at the target is significantly positively associated with sample size, sample size captures only a moderate amount of variance in this measure (estimate = 7.07, SE = 0.029, *p* < 0.001, *R* ^2^ = 0.44). Supplemental analyses indicate that the general associations between sample size and accuracy visualised in Figure 3 are largely consistent across different combinations of analysis parameters (Figure S4). Notably, a qualitatively different pattern of results emerged when only voxels yielding peak statistical values were used to generate accuracy metrics (Figure S2, Table S1).

### 3.2 Impact of Individual Univariate Analysis Choices

Table 1 reports analysis accuracy descriptive statistics for each considered univariate analysis factor. Regression analyses outperformed chi‐squared analyses in all considered accuracy metrics (target coverage, Dice, false positive rate). Chi‐squared analyses were more likely to yield significant results than regression analyses but were less likely to overlap with the target region. When considered alongside general analysis factors, the use of regression analyses positively predicted accuracy in terms of coverage (*t* = 48.13, *p* < 0.001, AIC change = 2304), Dice (*t* = 69.04, *p* < 0.001, AIC change = 4721), and false positive rate (*t* = −56.22, *p* < 0.001, AIC change = 3139).

**TABLE 1:** Analysis accuracy across different analysis design factors.

	Sample size	Volume	Per Sig	Per Hit	Results size	Dice	Coverage	FP rate
Stroke Side								
Unselected	612 (285)	34.0 (8.1)	69.30%	73.04%	140.5 (182.7)	0.04 (0.07)	**0.471 (0.424)**	0.967 (0.079)
L	235 (92)	27.2 (4.9)	68.11%	70.99%	54.3 (70.3)	**0.07 (0.09)**	0.376 (0.396)	**0.949 (0.100)**
R	323 (123)	38.8 (8.2)	58.71%	71.55%	91.7 (126.0)	0.05 (0.08)	0.403 (0.405)	0.961 (0.086)
Unilateral	539 (237)	32.7 (7.7)	68.99%	71.87%	136.1 (178.9)	0.04 (0.07)	0.452 (0.420)	0.968 (0.076)
Scan Type								
Unselected	625 (223)	35.8 (4.0)	71.73%	72.50%	133.9 (176.0)	0.045 (0.071)	**0.457 (0.424)**	0.970 (0.068)
CT	506 (179)	39.6 (4.3)	72.19%	71.98%	122.9 (163.8)	0.046 (0.072)	0.447 (0.422)	0.969 (0.068)
MR	130 (43.2)	20.2 (1.3)	49.50%	71.08%	51.6 (77.8)	**0.071 (0.098)**	0.359 (0.373)	**0.933 (0.131)**
Stroke Number								
Unselected	490 (282)	33.4 (8.3)	66.73%	72.55%	116.4 (162.1)	**0.050 (0.078)**	**0.440 (0.417)**	0.963 (0.083)
First Time Only	437 (237)	33.7 (8.5)	66.61%	71.52%	113.6 (159.0)	0.059 (0.077)	0.432 (0.415)	0.963 (0.085)
Analysis Type								
Chi Squared	464 (262)	33.6 (8.4)	73.09%	68.71%	131.5 (144.6)	0.028 (0.043)	0.390 (0.407)	0.982 (0.042)
Regression	464 (262)	33.6 (8.4)	65.25%	72.87%	110.9 (164.1)	**0.055 (0.083)**	**0.447 (0.417)**	**0.958 (0.090)**
Overlap Threshold								
> 0	464 (262)	33.6 (8.4)	87.68%	99.83%	165.5 (188)	**0.083 (0.094)**	**0.698 (0.328)**	**0.940 (0.102)**
> 10	464 (262)	33.6 (8.4)	71.11%	66.67%	110.9 (140.9)	0.029 (0.048)	0.334 (0.395)	0.979 (0.058)
> 10%	464 (262)	33.6 (8.4)	41.20%	22.15%	14.7 (21.1)	0.014 (0.047)	0.053 (0.173)	0.985 (0.063)
Multiple Comparison Corrections								
Bonferroni	464 (262)	33.6 (8.4)	68.97%	72.65%	89.5 (94.0)	0.040 (0.051)	0.446 (0.417)	0.974 (0.052)
FDR	464 (262)	33.6 (8.4)	71.22%	70.44%	188.6 (214.9)	0.024 (0.038)	**0.448 (0.424)**	0.985 (0.039)
Permutation	464 (262)	33.6 (8.4)	57.55%	73.70%	34.6 (52.9)	**0.106 (0.121)**	0.399 (0.397)	**0.908 (0.137)**
Lesion Volume Corrections								
Regression	464 (262)	33.6 (8.4)	68.04%	70.80%	129.0 (217.6)	0.055 (0.086)	0.435 (0.419)	0.959 (0.090)
None	464 (262)	33.6 (8.4)	70.19%	68.0%	118.4 (144.5)	0.042 (0.073)	0.421 (0.421)	0.968 (0.079)
DTLVC	464 (262)	33.6 (8.4)	57.50%	81.21%	80.4 (92.7)	**0.071 (0.089)**	**0.493 (0.407)**	**0.944 (0.102)**

*Note:* Value means and standard deviations (in parentheses) are presented for all univariate analyses employing each design factor. Sample size reports the mean number of patients included in analyses and volume reports the average lesion volume within this sample. Per Sig reports the proportion of analyses which yielded significant results. Per Hit reports the proportion of significant results which overlapped with the underlying anatomical target. Dice, Coverage, and FP rate report the mean accuracy of analyses yielding significant results in each category. Analysis factors yielding the highest accuracy (in terms of dice and target coverage) are highlighted in red.

### 3.3 Impact of Sample Inclusion Criteria Choices

Samples including all lesions (regardless of location) had the highest target coverage while samples including only left hemisphere patients yielded the highest Dice coefficient and lowest false positive rate (Table 1). When stroke side categories were added to the regression models predicting accuracy from general factors (described above), employing all lesions (regardless of location) significantly improved target coverage (*t* = 10.52, *p* < 0.001, AIC change = 109), Dice (*t* = 5.96, *p* < 0.001, AIC change = 34), and false positive rate (*t* = −8.62, *p* < 0.001, AIC change = 72). Including only right hemisphere lesions significantly improved analysis false positive rate (*t* = −8.36, *p* < 0.001, AIC change = 68). No other lesion location criteria significantly improved target coverage, Dice, or false positive rate.

Analyses including all patients, regardless of imaging modality, yielded the highest target coverage, while analyses using only MR resulted in the highest Dice and lowest false positive rate. When scan type inclusion criteria were incorporated into regression models predicting accuracy, using only CT scans improved analysis accuracy in terms of target coverage (*t* = 9.914, *p* < 0.001, AIC change = 96), Dice (*t* = 4.733, *p* < 0.001, AIC change = 21), and false positive rate (*t* = −3.31, *p* < 0.001, AIC change = 9). Analyses which used MR only also yielded improved false positive rates (*t* = −18.88, *p* < 0.001, AIC change = 354) relative to other analyses. Notably, MR‐only analyses had smaller sample sizes and were significantly more likely to produce false negative results (see Table 1). However, in cases where MR analyses did yield significant results, they produced comparatively high Dice and low false positive rates.

Including all lesions (regardless of lesion numerosity) yielded higher target coverage than first‐time stroke samples, but Dice and false positive rate were not different between these groups. Including the number of strokes for each individual as a covariate did not significantly add to models predicting target coverage from general analysis factors (Coverage: *t* = 0.374, *p* = 0.708, AIC change = 2). However, using all lesion samples (rather than first stroke only) was associated with improved Dice (*t* = 3.99, *p* < 0.001, AIC change = 14).

Overall, using all lesions (regardless of location), employing only CT‐derived lesions, and including all patients (regardless of stroke numerosity) were the best‐performing analysis choices.

### 3.4 Impact of Analysis Parameter Choices

Analyses which did not employ a minimum overlap threshold significantly outperformed those employing a minimum overlap threshold in terms of Dice, target overlap, and false positive rate. This finding cannot be a secondary consequence of increased results cluster sizes, as the use of no minimum overlap threshold positively correlated with both target coverage (*t* = 270.29, *p* < 0.001, AIC change = 113,469) and Dice (*t* = 378.36, *p* < 0.001, AIC change = 64,187), and negatively correlated with false positive rate (*t* = −162.19, *p* < 0.001, AIC change = 25,026) when general analysis factors were controlled for. No other overlap threshold criteria significantly improved target coverage, Dice, or false positive rate.

FWER permutation corrections yielded the highest Dice and lowest false positive rate, while FDR corrections produced the best target coverage. Notably, analyses using FDR corrections produced clusters which were much larger than those produced by analyses using FWER permutation or Bonferroni corrections. When general analysis factors (including the size of the results cluster) were controlled for, the use of FDR corrections predicted worse target coverage (*t* = −103.77, *p* < 0.001, AIC change = 10,543), worse Dice (*t* = −114.33, *p* < 0.001, AIC change = 12,743), and worse false positive rates (*t* = 88.62, *p* < 0.001, AIC change = 7733). Conversely, the use of FWER permutations predicted improved coverage (*t* = 52.18, *p* < 0.001, AIC change = 2706), Dice (*t* = 209.92, *p* < 0.001, AIC change = 40,625), and false positive rate (*t* = −186.37, *p* < 0.001, AIC change = 32,547) when general analysis factors were considered. The use of Bonferroni corrections predicted better target coverage (*t* = 51.24, *p* < 0.001, AIC change = 2611), but lower Dice (*t* = −59.55, *p* < 0.001) and higher false positive rates (*t* = 66.28, *p* < 0.001) when general analysis factors were controlled for. For this reason, FWER permutation corrections were selected as the best‐performing multiple comparison correction option.

The use of DTLVC lesion volume corrections yielded the best analysis accuracy in terms of both Dice and target coverage. The use of DTLVC corrections predicted improved analysis accuracy (over and above general analysis factors) in terms of both target coverage (*t* = 94.22, *p* < 0.001, AIC change = 1045), Dice (*t* = 48.77, *p* < 0.001, AIC change = 2655), and false positive rate (*t* = −38.94, *p* < 0.001, AIC change = 1509). Regressing out lesion volume did not improve target coverage, but predicted improved Dice (*t* = 5.79, *p* < 0.001, AIC change = 32) and false positive rate (*t* = −2.614, *p* = 0.008, AIC change = 5). DTLVC corrections were therefore selected as the best‐performing lesion volume correction.

Within analysis parameter factors, employing DTLVC lesion volume corrections, using FWER permutation corrections for multiple comparisons, and not using minimum lesion overlap analysis inclusion thresholds were found to yield the most accurate results.

### 3.5 Best‐Performing Models

Univariate analyses employing the best‐performing combination of analysis type, sample inclusion criteria, and analysis parameters (described above) were evaluated relative to analyses using other factor combinations. Of the 667 analyses that were run using the best‐performing analysis design, 83.5% yielded significant results 99.6% of which overlapped with the target. These significant analyses yielded an average coverage of 68.4% (SD = 31.9%, range = 0%–100%), an average Dice of 0.21 (SD = 0.12, range = 0–0.59), and an average false positive rate of 84.0% (SD = 12.2%, range = 19.8%–100%). Each of these scores was significantly higher within the best‐performing model relative to all other analysis designs (percent significant: X^2^ = 44.4, *p* < 001; percent hits: X^2^ = 263.4, *p* < 0.001; Coverage: *t* = 17.3, *p* < 0.001, Dice: *t* = 27.2, p < 0.001; false positive rate: *t* = 23.59, *p* < 0.001). Notably, mean coverage was 1.57 times higher, dice was 4.25 times higher, and false positive rate was 0.87 times lower within significant models using the best combination of factors relative to all other models.

### 3.6 Multivariate Analysis Simulation Results

**FIGURE 4:**
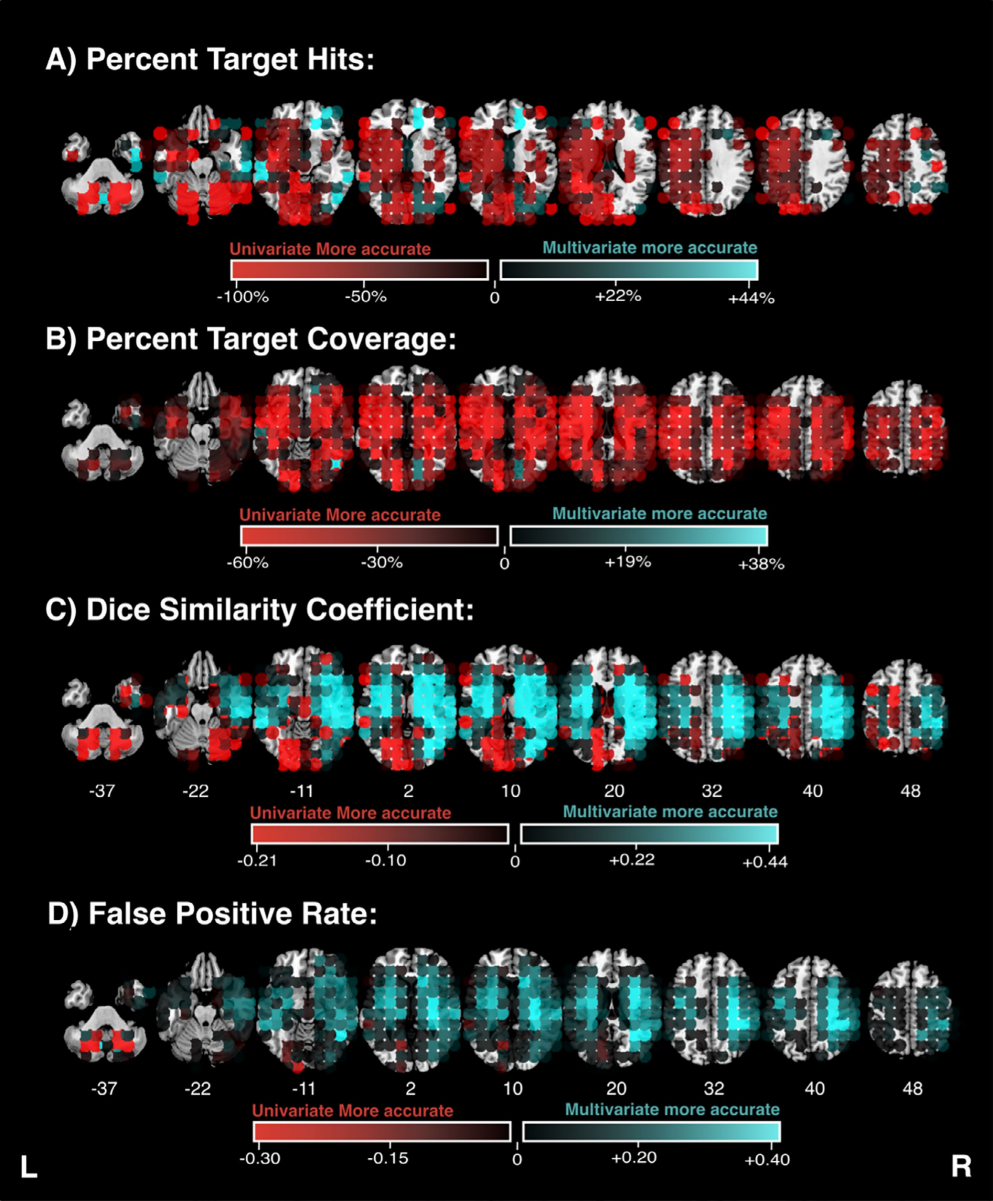
A comparison of lesion‐symptom mapping accuracy between multivariate (SCCAN) and univariate analysis approaches. Lesion‐symptom mapping accuracy across different anatomical target areas. Colour denotes mean accuracy for all analyses employing each defined anatomical target. Accuracy is quantified in terms of percent of analyses which yielded significant results overlapping with the target (Panel B), the percent of target voxels which were identified as significant (Panel C), and dice similarity coefficient between significant voxels and the underlying anatomical target (Panel D). L = left, R = right.

60.7% (*n* = 3665) of the potential multivariate analysis designs met criteria for inclusion in the simulation analysis. These accuracy data were compared with those of the univariate analyses using identical sample inclusion criteria, analysis parameters, and target ROIs. Multivariate analyses yielded a lower proportion of significant results relative to matched univariate analyses (63.0% vs. 79.8%, X^2^ = 2916.4, *p* < 0.001), and these results were less likely to overlap with the underlying target (44.2% target hits vs. 52.7%, X^2^ = 52.5, *p* < 0.001). Multivariate analyses yielded higher Dice relative to univariate analyses (0.068 vs. 0.025, *t*(2459.4) = 16.82, *p* < 0.001) and lower false positive rates (93.6% vs. 98.5%, *t*(2393.8) = −18.243, *p* < 0.001), but produced lower target overlap (0.15 vs. 0.24, *t*(4652.1) = −13.057, *p* < 0.001). These relationships remained consistent across different considered analysis designs (Tables 2 and 3). Accuracy differences between SCCAN and univariate approaches varied across different target ROIs (Figure 4), with the largest improvements in Dice coefficient and false positive rate in multivariate relative to univariate analyses present in areas with the most lesion coverage.

**TABLE 2:** SCCAN versus regression (univariate) lesion‐symptom mapping accuracy across different analysis factors.

	Per Sig	Per Hit	Results size	Dice	Coverage	FP rate
Stroke Side						
L	60.3% (−13.9)	46% (−4.3)	15.0 (−25.7)	0.069 (+0.035)	0.146 (−0.062)	0.945 (−0.036)
R	64.9% (−17.4)	43.1% (−12.8)	25.7 (−46.5)	0.067 (+0.042)	0.153 (−0.115)	0.928 (−0.064)
Overlap Threshold						
> 10	70.2% (−26.0)	61.7% (−6.6)	35.7 (−43.3)	0.088 (+0.053)	0.228 (−0.107)	0.931 (−0.048)
> 10%	55.8% (−4.7)	22.5% (−6.8)	3.6 (−20.3)	0.044 (+0.028)	0.053 (−0.035)	0.944 (−0.047)
Lesion Volume Corrections						
Regression	63.8% (−11.6)	39.8% (−15.6)	33.9 (−10.2)	0.071 (+0.035)	0.158 (−0.091)	0.928 (−0.050)
None	62.1% (−19.4)	48.8% (−2.3)	8.71 (−61.7)	0.065 (+0.045)	0.142 (−0.090)	0.945 (−0.043)

*Note:* Value means (for SCCAN analyses) are presented alongside the difference between SCCAN and univariate regression performance (in parentheses). Positive values represent cases in which values in SCCAN analyses were higher relative to univariate analyses and negative values represent cases in which SCCAN values were lower. Per Sig reports the proportion of analyses which yielded significant results. Per Hit reports the proportion of significant results which overlapped with the underlying anatomical target. Dice and Coverage report the mean accuracy of analyses yielding significant results in each category.

**TABLE 3:** SCCAN versus chi‐squared (univariate) lesion‐symptom mapping accuracy across different analysis factors.

	Per Sig	Per Hit	Results size	Dice	Coverage	FP rate
Stroke Side						
L	60.3% (−17.5)	46% (−2.2)	15.0 (−36.9)	0.069 (+0.047)	0.146 (−0.053)	0.945 (−0.042)
R	64.9% (−23.9)	43.1% (−9.6)	25.7 (−78.2)	0.067 (+0.053)	0.153 (−0.099)	0.928 (−0.057)
Overlap Threshold						
> 10	70.2% (−28.6)	61.7% (−6.4)	35.7 (−77.2)	0.088 (+0.065)	0.228 (−0.116)	0.931 (−0.055)
> 10%	55.8% (−13.0)	22.5% (−3.2)	3.6 (−30.8)	0.044 (+0.033)	0.053 (−0.024)	0.944 (−0.050)

*Note:* Value means (for SCCAN analyses) are presented alongside the difference between SCCAN and univariate chi‐squared performance (in parentheses). Positive values represent cases in which values in SCCAN analyses were higher relative to univariate analyses and negative values represent cases in which SCCAN values were lower. Per Sig reports the proportion of analyses which yielded significant results. Per Hit reports the proportion of significant results which overlapped with the underlying anatomical target. Dice and Coverage report the mean accuracy of analyses yielding significant results in each category.

## 4 Discussion

We have described a comprehensive investigation into how analysis design factors impact the accuracy of lesion‐symptom mapping analyses. This large‐scale simulation design provides novel insight into how a wide range of plausible analysis designs, samples, and underlying anatomical targets may interact to modulate the sensitivity and specificity of lesion‐symptom mapping analyses. Across all simulated designs, lesion‐symptom mapping analysis accuracy was highly variable and substantially modulated by the specific parameters employed in each analysis. These results provide important practical guidance by highlighting differential sample inclusion, analysis parameter, and analysis type choices which can be employed by authors aiming to maximise the specificity or sensitivity of lesion‐symptom mapping analyses. Consistent with prior simulation studies of lesion data (e.g., Ivanova et al. 2021; Mirman et al. 2018; Moore, Demeyere, et al. 2023; Sperber and Karnath 2017), the results of the current study demonstrate that there is no single optimal study design but that each individual analysis choice has advantages and disadvantages which must be considered in the context of a study's individual aims.

### 4.1 Factors Which Reliably Improve or Reduce Accuracy in Lesion‐Symptom Mapping

Despite the wide variability in accuracy amongst individual design choices, the results highlight several key factors which were consistently associated with either reduced or improved accuracy. First, analyses with better lesion coverage generated better accuracy. In univariate analyses, percent coverage of target ROIs improved dramatically in samples with > 50 lesions at the target relative to analyses with fewer “impaired” patients. This effect was less pronounced when accuracy was measured in terms of Dice, but a clear improvement was notable in analyses that included > 150 “impaired” patients. Notably, this effect is likely responsible for the comparative improvement in accuracy documented in subcortical regions relative to cortical regions (e.g., Figure 2), as subcortical regions had better lesion coverage in this study (see Figure 1A). This finding builds upon past work that established the importance of large sample sizes in lesion‐symptom mapping studies (Lorca‐Puls et al. 2018; Moore, Demeyere, et al. 2023). From first principles, statistical power is optimal where regions are impacted in half of the included sample. In real‐world lesion‐symptom mapping analyses, the degree of lesion overlap at the target correlate is a function of the overall sample size and the number of patients within the sample who are impaired on the behavioural measure of interest.

Importantly, this finding accounts for why analyses employing CT imaging (rather than using MR imaging alone) yielded improved accuracy. While MR imaging has a higher sensitivity to lesion damage than CT (Brazzelli et al. 2009; Kloska et al. 2004), CT imaging is often available for a wider range of patients as it is often the first‐line clinical imaging modality and has fewer contraindications (Lövblad et al. 2015; Wardlaw, Keir, et al. 2004; Wardlaw, Seymour, et al. 2004). As behaviour is simulated, lesion sensitivity differences across modalities are not captured in this study, but differences in the samples included in studies due to imaging modality requirements are. Both in the present simulation study and in real‐world lesion‐symptom mapping studies, designs which include CT imaging generally include larger samples (de Haan and Karnath 2018; Moore, Demeyere, et al. 2023). Given that sample size and degree of lesion coverage were identified as critical determinants of analysis sensitivity and specificity, our study provides additional empirical support for the assertation that, where possible, lesion‐symptom studies can and should include lesions derived from CT imaging (de Haan and Karnath 2018; Moore, Demeyere, et al. 2023). We found a negligible impact of including patients based on multiple stroke lesions and a clear benefit of combining different neuroimaging modalities in lesion‐symptom mapping. These results suggest that future studies could employ more lenient inclusion criteria to maximise the number of patients included in analyses.

For the univariate analyses, approaches using direct total lesion volume controls (DLVTC) reliably outperformed other volume correction options in terms of Dice, target coverage, and false positive rate. In line with past work, regression‐based volume corrections were more accurate than analyses with no volume corrections but yielded less accurate results relative to DLVTC approaches (DeMarco and Turkeltaub 2018; Zhang et al. 2014). Past simulation work has theorised that regression‐based corrections may be too conservative for lesion‐symptom mapping because critical variability relating to true correlates may be regressed out alongside lesion volume (Sperber 2022). The results of our study align with this hypothesis, as DLVTC approaches are less conservative and were found to generate more accurate results. Notably, many popular lesion‐symptom mapping software toolkits do not offer DLVTC as a built‐in correction method. Given the demonstrated benefit of DLVTC approaches, researchers should aim to use toolkits which offer the option to use this approach, and developers should aim to integrate this option into new and existing lesion‐symptom mapping analysis packages.

Permutation corrections (FWER) resulted in the highest Dice and lowest false positive rate, while FDR corrections resulted in the highest target coverage (and the highest false positive rate). Notably, Mirman et al. (2018) developed an alternate cluster‐based FWER permutation correction approach which was found to outperform the FWER approach used here, making it a strong analytical option for future high‐quality lesion mapping studies. Employing a minimum voxel‐level inclusion criterion of 10% of the total sample was associated with reduced performance across all considered designs and accuracy measures. Studies using 10% overlap inclusion thresholds were less likely to generate significant results and, in cases where significant results were produced, were less accurate than the other tested cutoffs. Employing minimum overlap thresholds is intended to reduce spatial displacement of lesion‐symptom mapping results (Karnath et al. 2018; Sperber and Karnath 2017). Spatial bias is generally strongest within cortical regions, meaning that the inclusion of many subcortical targets in our analysis may have masked any potential accuracy benefit from employing stricter minimum overlap thresholds (Karnath et al. 2018; Sperber and Karnath 2017). The use of 10% overlap inclusion thresholds is also more commonly used in studies with smaller samples (< 50) relative to larger‐scale investigations which frequently opt for minimum inclusion cut‐offs which do not scale with sample size (e.g., > 3, > 5, > 10). It is plausible that using a 10% overlap inclusion threshold is more poorly suited to lesion‐symptom mapping studies with larger samples (> 100) relative to smaller samples (< 50), as the number of overlapping parcels needed to meet a percentage‐based cut‐off will increase as sample size increases. This possibility was not explicitly evaluated in our study, as our pre‐registered design prioritised generating results which are applicable to large‐scale studies meeting current minimum sample size recommendations (Moore, Demeyere, et al. 2023; Sperber et al. 2019). The results of the current study suggest that a 10% overlap inclusion criterion may not be optimal in lesion‐symptom mapping studies involving samples between 67 and 959 patients.

Within univariate analyses, chi‐squared approaches were consistently less accurate than regression‐based approaches. Chi‐squared approaches were more likely to generate significant results, but these results were less likely to contain the target region and were less accurate. We binarised simulated behavioural scores for chi‐squared analyses using a single threshold (0.50). It is unclear whether the results reported here would remain consistent across all potential impairment thresholds, but it seems plausible that approaches that constrain variability in input behavioural scores may be limited in their ability to precisely localise brain‐behaviour relationships (de Haan and Karnath 2018). There are also several alternative statistical approaches to handle binarised behavioural data, such as analyses using Liebermeister measures as the base statistical test (Rorden et al. 2007). Given that behavioural data from patient samples is often non‐parametric, it is important to evaluate what the optimal method for handling categorical and/or binary scores in lesion‐symptom mapping may be.

### 4.2 Key Differences From Previous Lesion‐Symptom Mapping Work

The results of our study are largely in line with previous work (e.g., Ivanova et al. 2021; Mirman et al. 2018; Moore, Demeyere, et al. 2023; Sperber and Karnath 2017), but there are several notable exceptions. First, analyses that did not employ a minimum overlap threshold generated more accurate results in terms of percent hits, Dice, target coverage, and false positive rate. This result is surprising as previous simulation work has concluded that applying a minimum overlap inclusion threshold improves lesion‐symptom mapping accuracy (Sperber and Karnath 2017). It is plausible that minimum overlap thresholds may differentially interact with other correction factors. For example, significance thresholds for analyses using some correction approaches (e.g., Bonferroni) may become stricter when minimum overlap thresholds are not used, as this would increase the number of tested voxels.

Analyses which employed only lesions derived from CT imaging consistently outperformed analyses which used a combination of MR and CT or MR only. This result is surprising for two reasons: first, because selecting based on imaging type reduces sample size (see Table 1), and second because MR is generally more sensitive to lesion damage (Brazzelli et al. 2009; Kloska et al. 2004). Within MR, specific modalities such as T2 and FLAIR are more well suited to lesion segmentation than others (e.g., T1) (de Haan and Karnath 2018; Moore, Demeyere, et al. 2023). Most of the data used in the current study came from UK stroke settings where MR imaging is generally only used in special cases where patients have small lesions which are difficult to localise using CT or are neurologically complex cases (e.g., comorbid neurological abnormalities) (Lövblad et al. 2015; Wardlaw, Keir, et al. 2004; Wardlaw, Seymour, et al. 2004). This sample difference is reflected in the finding that the MR imaging used in this study depicts smaller lesions with a qualitatively different spatial topography than CT imaging (see Group 20 vs. Group 22 in Figure S5). It is therefore plausible this distribution difference may have negatively impacted analysis accuracy, but it is unclear whether this effect would be expected to generalise to other simulated or real‐world lesion analyses.

### 4.3 Univariate Versus Multivariate Lesion‐Symptom Mapping Analyses

Multivariate analyses consistently outperformed univariate analyses in terms of Dice and false positive rate, but univariate analyses were reliably more accurate in terms of target coverage. This result is in line with past work indicating that there is no universal superiority of one analysis type over another, but that each analysis type offers specific advantages and weaknesses which must be considered in the context of individual study goals (Brazzelli et al. 2009).

Past simulation work which has reported that multivariate analyses consistently outperform univariate analyses has generally quantified accuracy in terms of measures such as Dice coefficient and results displacement distance (Mah et al. 2014; Pustina 2019). Our study demonstrates that these measures may not fully capture the performance of lesion‐symptom mapping analyses, because improvements in Dice scores were generally associated with decreases in target coverage. Results displacement is strongly associated with the size of significant voxel clusters (Moore, Demeyere, et al. 2023), meaning that displacement may appear to be higher in univariate versus multivariate analyses solely due to the comparatively larger significant voxel clusters generated by univariate analyses.

Additionally, lesion‐symptom mapping accuracy was often low even within the best‐performing univariate and multivariate analysis designs. This result indicates that neither multivariate nor univariate lesion‐symptom mapping analysis designs should be assumed to be perfectly accurate, as both analysis designs are susceptible to voxel‐wise false positives and misses arising from factors such as variable lesion overlap (e.g., statistical power), and spatial biases due to non‐random spatial distributions of lesion location determined by vascular territories (Kimberg et al. 2007; Mah et al. 2014; Moore, Demeyere, et al. 2023). Overall, these findings are in line with previous work reporting that multivariate and univariate approaches both have benefits and weaknesses, and that the optimal analysis type depends on the specific goals of individual analyses.

### 4.4 Sensitivity Versus Specificity in Lesion‐Symptom Mapping

Lesion‐symptom mapping, like any statistical approach, entails an inherent trade‐off between sensitivity and specificity. The results of the current study provide clear guidance to authors aiming to design lesion‐symptom mapping analyses which prioritise sensitivity or specificity (Table 4) while also highlighting the trade‐offs associated with each approach. This aligns with previous work recommending that the exact statistical approach taken in lesion‐symptom mapping studies should depend on the precise research question being explored (DeMarco and Turkeltaub 2018). Studies which aim to maximise sensitivity prioritise detecting correlates (if they exist) over minimising voxel‐wise false positives. In this case, it is methodologically valid for authors to adopt more liberal correction approaches that maximise the probability of generating results which overlap with the underlying target (e.g., univariate analysis designs, FDR corrections, no corrections for lesion volume). However, this approach limits the degree to which the identified anatomy can be assumed to be functionally related to the deficit of interest due to its high voxel‐wise false positive rate. Studies prioritising sensitivity should therefore strictly limit theoretical interpretation and causal inferences pertaining to identified correlates, and clearly acknowledge the elevated risk of voxel‐wise false positives. Sensitivity‐driven analyses can instead focus on describing broad lesion patterns associated with the behaviour of interest and qualitatively comparing these patterns across patient groups. This approach may be most appropriate in cases where samples are small, and where impairments are rare and descriptive.

Conversely, studies which aim to prioritise specificity may adopt more conservative statistical approaches (e.g., multivariate analyses, permutation‐ corrections, DLVTC lesion volume corrections). Specificity‐driven studies can be more confident that identified regions are causally involved in the underlying function of interest, while acknowledging that some critical regions may be missed. This approach is ideal for studies aiming to make causal interpretations about identified neural correlates, because significant results arising from this more rigorous analytical approach provide stronger evidence in support of causal brain‐behaviour relationships than more liberal statistical approaches.

Sensitivity‐driven and specificity‐driven analysis designs are not mutually exclusive; indeed, it is feasible to use both analytical approaches in tandem. Correlates which remain significant in both sensitivity‐driven and specificity‐driven analytical designs can be confidently interpreted as being causally involved with the behaviour of interest (Ivanova et al. 2021). In cases where significant results do not emerge from strict, specificity‐driven analyses, the results of sensitivity‐driven analyses can be used to identify the presence of broader lesion patterns which may distinguish between behavioural groups of interest. This combined analytical approach is in line with past work suggesting that combining both multivariate and univariate lesion‐symptom mapping analyses within individual studies may be the most informative way to identify and understand the role of implicated neural correlates (Ivanova et al. 2021). The present study adds to this previous work by identifying a range of additional parameters, outside of univariate/multivariate approaches, which can be used to tailor analyses toward prioritising voxel‐wise sensitivity and specificity (Table 4).

**TABLE 4:** Summary guidance for analysis designs prioritising sensitivity and specificity, based on the results of this study.

	Prioritising sensitivity	Prioritising specificity
Example Questions	“What lesions are associated with deficit X?”	“What correlates support function X?”
	“Which areas are disconnected in group X?”	“Is deficit X more strongly linked to areas A or B?”
	“How are lesions different between groups A and B?”	“Is network A linked to deficit X?”
Inclusion Criteria		
Lesion Location	Do not restrict	Do not restrict
Imaging Modality	Do not restrict	Do not restrict
Stroke Numerosity	Do not restrict	Do not restrict
Analysis Parameters		
Statistical Test	Univariate Regression	Multivariate
Overlap Threshold	Do not restrict	Do not restrict
Multiple Comparisons	FDR	Permutation
Volume Correction	Not necessary	DTLVC

*Note:* Given that sample size and degree of lesion overlap at the target correlate emerged as key drivers of analysis accuracy, sample inclusion criteria for all studies should aim to maximise sample size. Supporting rationale (and caveats) for all other recommendations are discussed above.

Abbreviations: DTLVC, Direct Total Lesion Volume Control; FDR, False Discovery Rate.

### 4.5 Limitations

There are several factors that may modulate lesion‐symptom mapping accuracy which were not evaluated in this study. These include several common statistical tests (e.g., Liebermiester tests, *t*‐tests, support vector regression) as well as important factors (e.g., sample size) which were not explicitly manipulated in this study. Notably, SCCAN may not be comparable to all multivariate lesion‐symptom mapping approaches, as the statistical approach used in this analysis conceptually differs from other lesion‐symptom mapping approaches (Pustina et al. 2018). While other univariate and multivariate lesion‐symptom mapping analyses support voxel‐wise inferences (and multiple comparison corrections), SCCAN outputs a sparse predictive map (Pustina et al. 2018). For this reason, future work is needed to confirm whether the observed effects remain consistent in other multivariate lesion mapping approaches such as support vector regression.

Additionally, our design prioritised voxel‐level lesion‐symptom mapping approaches. It remains unclear whether the documented effects would also hold for disconnection‐based approaches such as network and tract‐level lesion‐symptom mapping. The key conclusions of this study are intended to provide guidance for lesion‐symptom mapping studies which interpret brain‐behaviour relationships based on all significant voxels emerging from analyses. The reported relationships vary when accuracy is measured based on voxels yielding peak statistical values, and these relationships are detailed in the Supporting Information.

Our study simulated behavioural deficits based on the degree to which real lesions overlap with single, spatially continuous brain ROIs. It is not possible to know whether the documented effects would remain consistent in cases where behaviour is supported by multiple, spatially distant regions or the connections between these regions. The target ROIs we used here are also not naturalistic as they were represented by voxel spheres which would not be expected to align with real‐world functional ROI boundaries, or the overlap topographies present in lesion overlays. This approach was followed to maximise coverage of brain regions while standardising the size and shape of underlying targets. However, it may be more ecologically valid to use atlas‐derived regions to simulate behavioural impairments. Future studies should aim to evaluate whether consistent results are found when more realistic potential underlying neural correlates are considered (e.g., functional ROIs). Previous work has demonstrated that adding noise to simulated behavioural scores can dramatically reduce the accuracy of lesion‐symptom mapping analyses (Ivanova et al. 2021). We did not include this manipulation, and therefore cannot evaluate whether different lesion‐symptom mapping analysis types are more susceptible to the presence of behavioural noise.

## 5 Conclusions

We have presented a comprehensive investigation into how individual analysis choices modulate results accuracy in lesion‐symptom mapping studies. We identified several key analytical choices which were universally associated with improved lesion‐symptom mapping accuracy. However, the results of our study clearly illustrate that the optimal analysis design is ultimately dependent on the specific goals of individual studies. For this reason, it is important that lesion‐symptom mapping analysis parameters are explicitly selected to prioritise results sensitivity or specificity, and for any resultant significant voxel clusters to be interpreted with this design's strengths and limitations in mind. We believe that this practice, when applied systematically, has the potential to improve both the quality and accuracy of lesion‐symptom mapping analyses as well as improving the reliability of theoretical inferences made based on lesion‐symptom mapping results.

## Author Contributions

In line with the CReDiT taxonomy, M.J.M. was responsible for conceptualisation, software, data curation, formal analysis, methodology, visualisation, and writing (original draft). C.R. was responsible for writing (review and editing). G.A.R. was responsible for resources and writing (review and editing). J.B.M. was responsible for supervision and writing (review and editing). N.D. was responsible for supervision, resources, and writing (review and editing).

## Funding

M.J.M. is supported by an Australian Research Council Discovery Early Career Research Award (DE240100327). N.D. (Advanced Fellowship NIHR302224) is funded by the National Institute for Health Research (NIHR). The views expressed in this publication are those of the author(s) and not necessarily those of the NIHR, NHS or the UK Department of Health and Social Care.

## Conflicts of Interest

The authors declare no conflicts of interest.

## Supporting information


**Figure S1:** Lesion overlay plots for the subsample of participants with imaging collected at chronic timepoints (Panel A, *n* = 57) versus the full sample (Panel B). Colour denotes number of overlapping lesions at each location. MNI slices 37–58 are presented.
**Figure S2:** The relationship between key sample/results characteristics and degree of agreement between peak voxels and target voxels. Considered analysis factors are listed across the *x*‐axis, and different accuracy measures (Coverage, Dice, false positive (FP) rate) are on the *y*‐axis. The coefficients and significance for each factor within the described linear mixed model analyses are listed in the top left of each panel, and each panel depicts the best‐fit general linear model for each individual factor. Est = linear mixed model estimate value, se = standard error. All parameters, except for number of lesions impacting the target in the Coverage mode (*p* = 0.839), have *p*‐values < 0.001.
**Table S1:** Analysis accuracy (quantified relative to peak voxels) across different analysis design factors. Value means and standard deviations (in parentheses) are presented for all univariate analyses employing each design factor. Dice, Coverage, and FP rate report the mean accuracy of analyses yielding significant results in each category. Analysis factors yielding the highest accuracy (in terms of dice and target coverage) are highlighted in red.
**Figure S3:** The relationship between Dice coefficients and target percent coverage across all simulated univariate analyses which yielded significant results.
**Figure S4:** The relationship between sample size and accuracy across different analysis parameters. Sample size group is presented on the *x*‐axis and accuracy measures (Percent coverage, Dice, and false positive rate) are presented on the *y*‐axis. Group means and standard error ranges are plotted for each group. The mean accuracy is plotted as a grey dashed line for reference. These analyses indicate that the relationship between sample size and accuracy at the group level (Figure 2) is largely preserved across different analytical choices. Regres. = Regression, Chisq. = chi‐squared. Min. Over. = Minimum overlap threshold. MC = multiple comparison. Perm = FWER permutation, Bonf = Bonferroni, Vol = Volume.
**Figure S5:** Lesion overlays for each of the possible 24 patient groups. Colour denotes the number of lesions impacting each area. MNI axial slices between *X* = −40–58 are shown.

## Data Availability

The data that support the findings of this study are openly available in Open Science Framework at https://osf.io/6kcha/overview.
