# Supplementary material for: Methodological Choices Strongly Modulate the Sensitivity and Specificity of Lesion‐Symptom Mapping Analyses
Source: Hum Brain Mapp. 2026 Jul 30;47(11):e70619. doi: 10.1002/hbm.70619 (PMC13421372; doi:10.1002/hbm.70619)
Supplement: Supplementary file 1 — Figure S1: Lesion overlay plots for the subsample of participants with imaging collected at chronic timepoints (Panel A, n = 57) versus the full sample (Panel B). Colour denotes number of overlapping lesions at each location. MNI slices 37–58 are presented. Figure S2: The relationship between key sample/results characteristics and degree of agreement between peak voxels and target voxels. Considered analysis factors are listed across the x‐axis, and different accuracy measures (Coverage, Dice, false positive (FP) rate) are on the y‐axis. The coefficients and significance for each factor within the described linear mixed model analyses are listed in the top left of each panel, and each panel depicts the best‐fit general linear model for each individual factor. Est = linear mixed model estimate value, se = standard error. All parameters, except for number of lesions impacting the target in the Coverage mode (p = 0.839), have p‐values < 0.001. Table S1: Analysis accuracy (quantified relative to peak voxels) across different analysis design factors. Value means and standard deviations (in parentheses) are presented for all univariate analyses employing each design factor. Dice, Coverage, and FP rate report the mean accuracy of analyses yielding significant results in each category. Analysis factors yielding the highest accuracy (in terms of dice and target coverage) are highlighted in red. Figure S3: The relationship between Dice coefficients and target percent coverage across all simulated univariate analyses which yielded significant results. Figure S4: The relationship between sample size and accuracy across different analysis parameters. Sample size group is presented on the x‐axis and accuracy measures (Percent coverage, Dice, and false positive rate) are presented on the y‐axis. Group means and standard error ranges are plotted for each group. The mean accuracy is plotted as a grey dashed line for reference. These analyses indicate that the relationship between sa [file HBM-47-e70619-s001.docx]

**Supplementary Materials:**

*Lesion size and distribution are comparable between acute and non-acute scans*

This study employs a combination of routine clinical imaging obtained acutely (<30 days post-stroke, n = 909) and at later (chronic) timepoints (>30 days post stroke, n = 50). Supplementary analyses were conducted to evaluate whether these imaging samples were comparable in terms of lesion severity and lesion distribution. Lesion volume was not significantly different between acute imaging (mean volume = 34.8 cm^3^, SD = 53.3) and chronic imaging (mean volume = 47.5 cm^3^, SD = 61.2) (t-test, t(63.31) = -1.517, p = 0.134). The subset of lesions collected at chronic timepoints (n = 57) exhibited a qualitatively similar lesion overlay to the full sample (Supplementary Figure 3). Specifically, in both the full sample and this subsample lesion overlay was highest in subcortical regions within the right MCA territory. This pattern was mirrored in the left hemisphere in both samples, but with a lower degree of overlap.


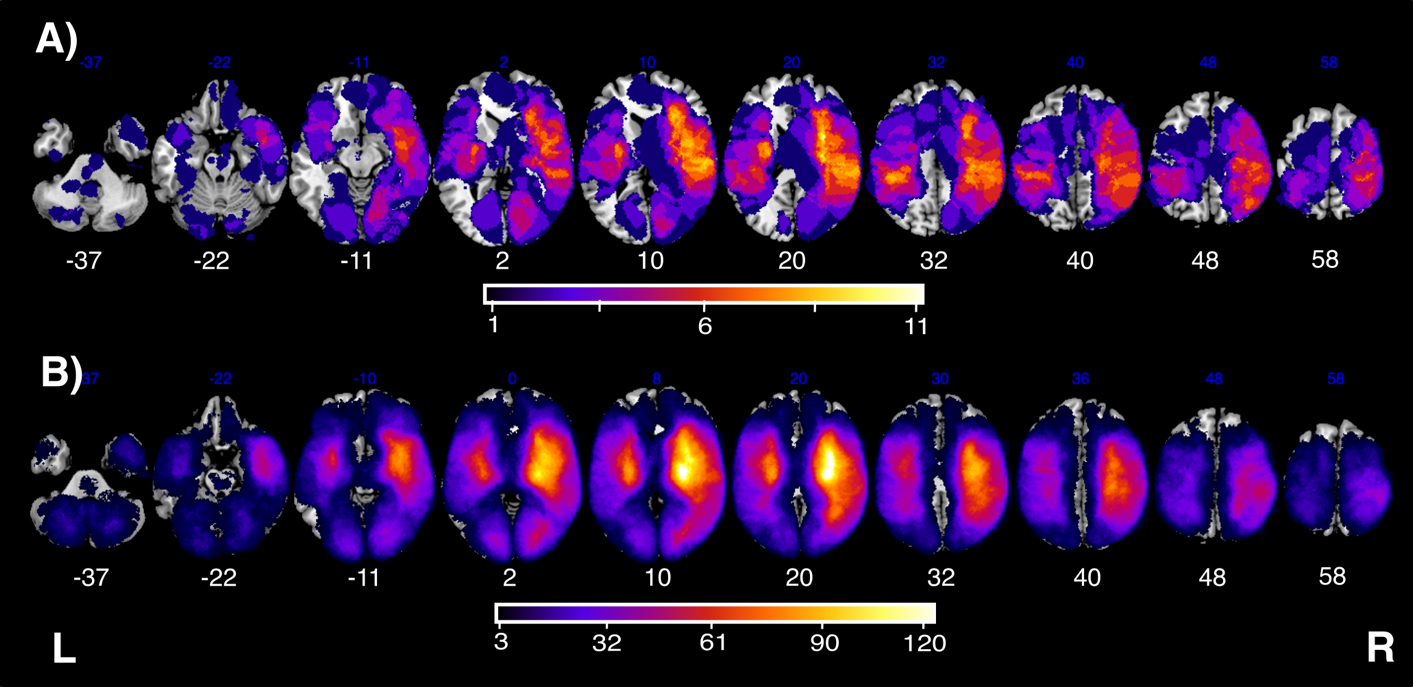


***Supplementary Figure 1:*** *Lesion overlay plots for the subsample of participants with imaging collected at chronic timepoints (Panel A, n = 57) versus the full sample (Panel B). Colour denotes number of overlapping lesions at each location. MNI slices -37 – 58 are presented.*

*Accuracy relationships differ when only voxels yielding peak statistical values are considered:*

Previous research has indicated that the precision of lesion mapping analyses is increased when results are interpreted as a function of voxel-level statistical values rather than binarizing results as significant or non-significant (Mah et al., 2014; Moore, Jenkinson, et al., 2023). Specifically, studies have found that accuracy increases when only voxels yielding peak statistical values are considered (Mah et al., 2014; Moore, Jenkinson, et al., 2023).

A qualitatively different pattern emerged when accuracy was quantified relative to peak voxels rather than all significant voxels. Dice and percent coverage were lower when only peak voxels were considered relative to when all significant voxels were considered (Dice Statistics: mean peak dice = 0.002, overall dice = 0.050, t(291821) = -295.2, p < 0.001; Coverage statistics: mean peak coverage = 0.028, overall coverage = 0.436, t(277110) = -481.92 p < 0.001). However, false positive rate was improved in peak voxels (mean FP rate = 0.717) compared with all voxels (mean FP rate = 0.963) (t(270819) = -271.04, p < 0.001).

Across all univariate analyses yielding significant results, multivariate regression revealed that results accuracy (in terms of coverage, Dice, and false positive rate) was significantly modulated by sample size, average sample lesion volume, number of lesions impacting the target, and significant results cluster size (Dice Model R^2^ = 0.025, Percent Coverage Model R^2^ = 0.136, False Positive Rate Model R^2^ = 0.025). All parameters, with the exception of number of lesions impacting the target in the Coverage mode (p = 0.839), have p-values <0.001.

When individual inclusion criteria are considered, parameters which reduced the number of voxels tested in individual analyses resulted in the highest peak voxel accuracy (Supplementary Table 1). Regression analyses generated the most accurate peak coverage and Dice scores, and chi squared analyses yielded the lowest false positive rate. Using no minimum overlap threshold resulted in the highest peak Dice and coverage, while using a cut-off of 10% generated the lowest false positive rate. Bonferroni corrections yielded the best peak Dice and coverage, while permutation corrections resulted in the lowest peak false positive rate. Regression volume corrections lead to the highest Dice, DLVTC lead to the best coverage, and using no volume correction yielded the best false negative rate.

Overall, the pattern of observed results is qualitatively different when accuracy is quantified relative to peak voxels. In many cases, the observed relationships between parameters and peak voxels sharply contrast with factors which are known to improve lesion mapping power and accuracy. For example, the most effective way to improve the accuracy of peak voxels appears to be to test as few voxels as possible from as small a sample as possible. Better statistical power, as represented by increased sample size and lesion overlap at the underlying target, lead to worse peak voxel accuracy scores. Notably, real-world lesion mapping studies generally interpret the anatomy of all significant voxels rather than restricting interpretation to peak voxel coordinates (Bates et al., 2003; de Haan & Karnath, 2018; Moore, Demeyere, et al., 2023). However, these observed relationships between analysis parameters may provide useful insights for the design of future studies aimed at interpreting only peak voxel anatomy.


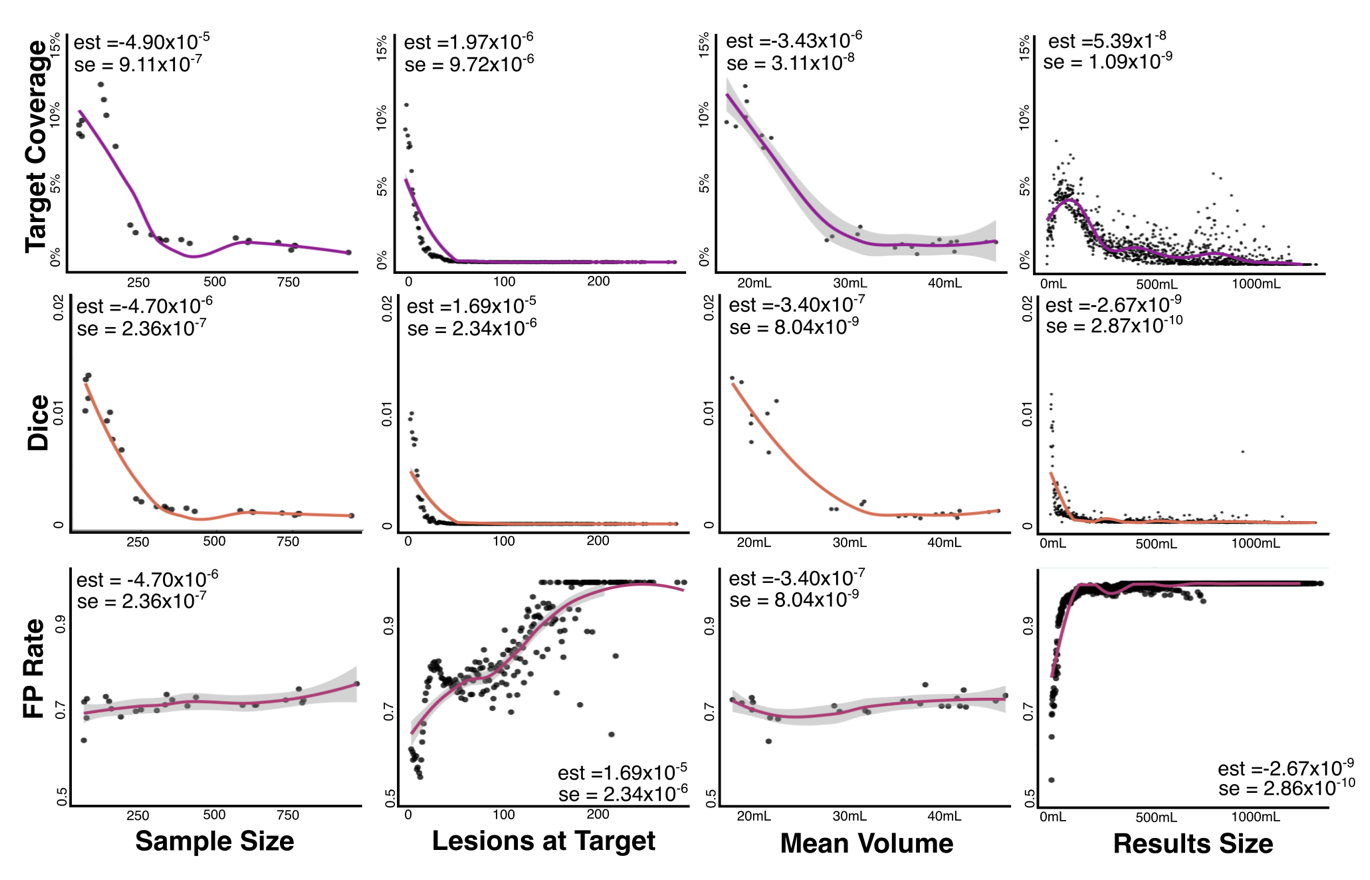
=

***Supplementary Figure 2:*** *The relationship between key sample/results characteristics and degree of agreement between peak voxels and target voxels. Considered analysis factors are listed across the x-axis, and different accuracy measures (Coverage, Dice, false positive (FP) rate) are on the y-axis. The coefficients and significance for each factor within the described linear mixed model analyses are listed in the top left of each panel, and each panel depicts the best-fit general linear model for each individual factor. Est = linear mixed model estimate value, se = standard error. All parameters, except for number of lesions impacting the target in the Coverage mode (p = 0.839), have p-values <0.001.*


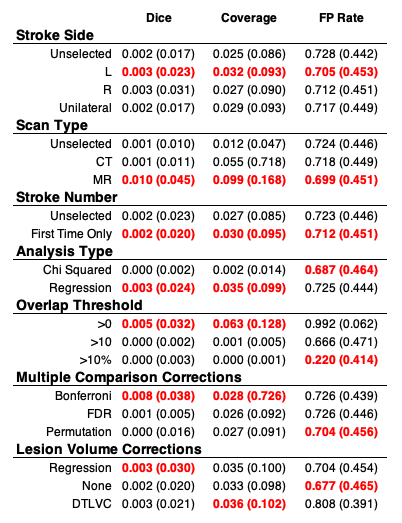


***Supplementary Table 1:*** *Analysis accuracy (quantified relative to peak voxels) across different analysis design factors. Value means and standard deviations (in parentheses) are presented for all univariate analyses employing each design factor. Dice, Coverage, and FP rate report the mean accuracy of analyses yielding significant results in each category. Analysis factors yielding the highest accuracy (in terms of dice and target coverage) are highlighted in red.*


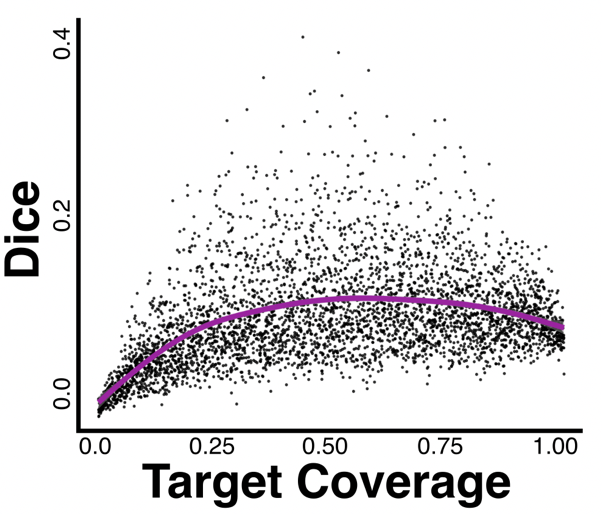


***Supplementary Figure 3:*** *The relationship between Dice coefficients and target percent coverage across all simulated univariate analyses which yielded significant results.*


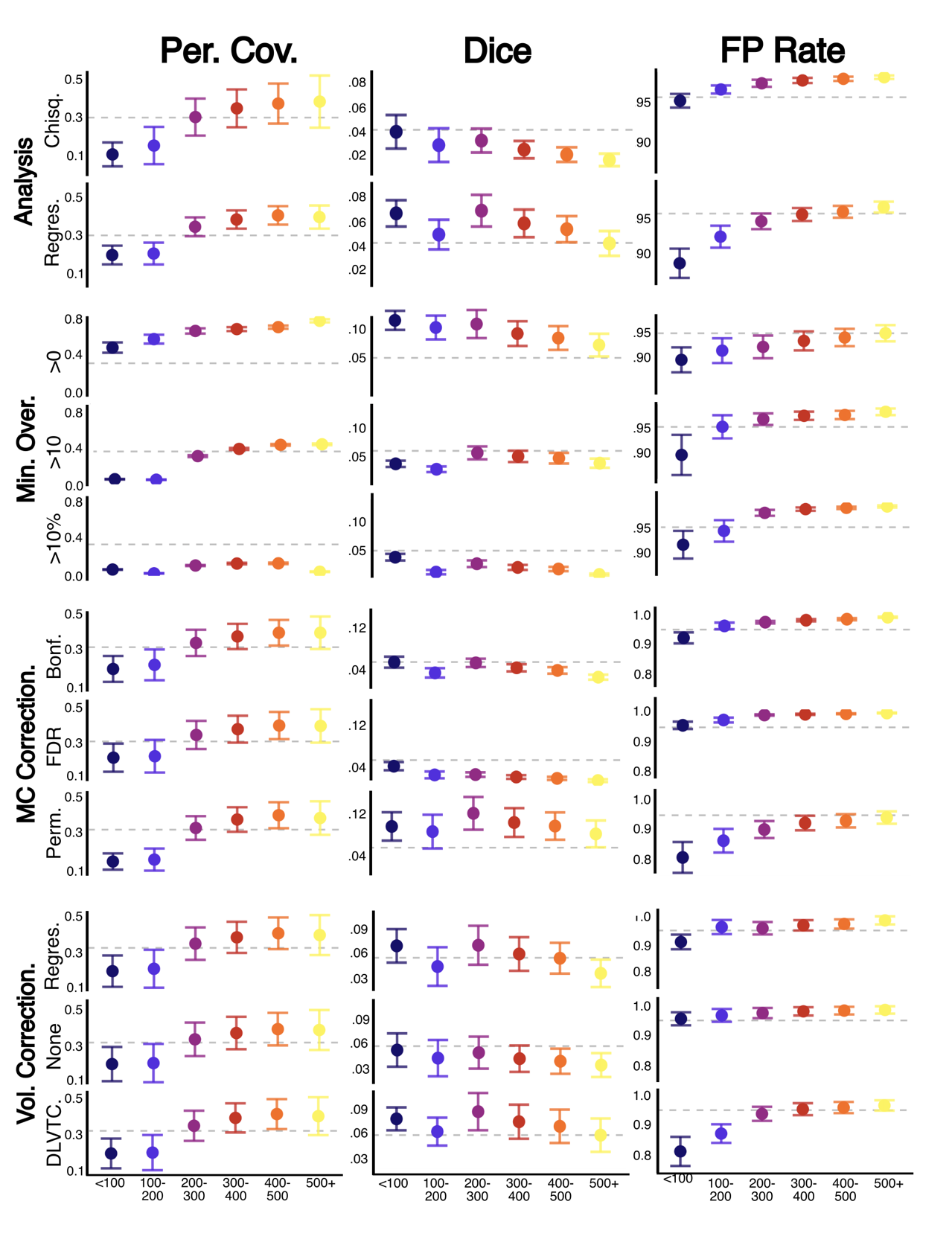

***Supplementary Figure 4****: The relationship between sample size and accuracy across different analysis parameters. Sample size group is presented on the x-axis and accuracy measures (Percent coverage, Dice, and false positive rate) are presented on the y-axis. Group means and standard error ranges are plotted for each group. The mean accuracy is plotted as a grey dashed line for reference. These analyses indicate that the relationship between sample size and accuracy at the group level (Figure 2) is largely preserved across different analytical choices. Regres. = Regression, Chisq. = chi-squared. Min. Over. = Minimum overlap threshold. MC = multiple comparison. Perm = FWER permutation, Bonf = Bonferroni, Vol = Volume.*

Targ


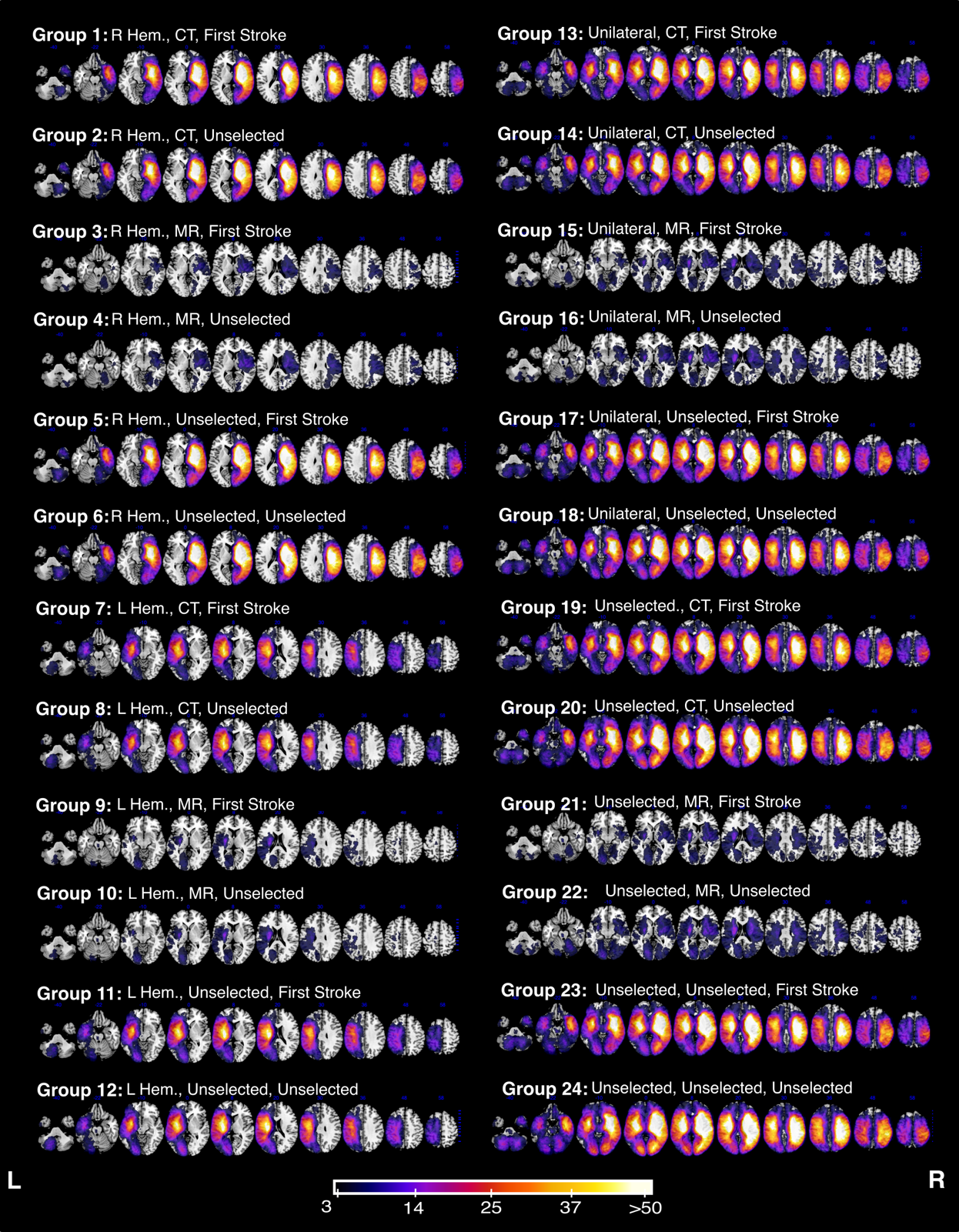


***Supplementary Figure 5:*** *Lesion overlays for each of the possible 24 patient groups. Colour denotes the number of lesions impacting each area. MNI axial slices between X = -40 – 58 are shown.*
